# Shape shifter: redirection of prolate phage capsid assembly by staphylococcal pathogenicity islands

**DOI:** 10.1038/s41467-021-26759-x

**Published:** 2021-11-04

**Authors:** N’Toia C. Hawkins, James L. Kizziah, José R. Penadés, Terje Dokland

**Affiliations:** 1 Department of Microbiology, University of Alabama at Birmingham, Birmingham, AL USA; 2 Center for Molecular Bacteriology and Infection, Imperial College London, London, UK

**Keywords:** Bacteriophages, Bacterial genetics, Cryoelectron microscopy

## Abstract

*Staphylococcus aureus* pathogenicity islands (SaPIs) are molecular parasites that hijack helper phages for their transfer. SaPIbov5, the prototypical member of a family of *cos* type SaPIs, redirects the assembly of ϕ12 helper capsids from prolate to isometric. This size and shape shift is dependent on the SaPIbov5-encoded protein Ccm, a homolog of the ϕ12 capsid protein (CP). Using cryo-electron microscopy, we have determined structures of prolate ϕ12 procapsids and isometric SaPIbov5 procapsids. ϕ12 procapsids have icosahedral end caps with *T*_end_ = 4 architecture and a *T*_mid_ = 14 cylindrical midsection, whereas SaPIbov5 procapsids have *T* = 4 icosahedral architecture. We built atomic models for CP and Ccm, and show that Ccm occupies the pentameric capsomers in the isometric SaPIbov5 procapsids, suggesting that preferential incorporation of Ccm pentamers prevents the cylindrical midsection from forming. Our results highlight that pirate elements have evolved diverse mechanisms to suppress phage multiplication, including the acquisition of phage capsid protein homologs.

## Abstract (web summary)

Phage-inducible chromosomal islands (PICIs) are a group of mobile genetic elements that hijack the replication and assembly machinery of helper bacteriophages. Here the authors describe a mechanism by which a group of PICIs from *Staphylococcus aureus* re-direct the assembly pathway of their helpers using a capsid protein homolog.

## Introduction

*Staphylococcus aureus* is an important human pathogen responsible for a broad spectrum of diseases, including life-threatening conditions such as bacteremia and sepsis^1^. *S. aureus* encodes a wide array of virulence factors that increase bacterial survival in the human host and that may increase the severity of the disease. Many of these virulence factors are encoded on mobile genetic elements (MGEs)—including plasmids, bacteriophages, and genomic islands—that are transferred horizontally between hosts^2^. Bacteriophages play an especially important role among the MGEs, since transduction by bacteriophages is the main mode of horizontal gene transfer in *S. aureus*^3,4^ All natural strains of *S. aureus* contain one or more prophages (integrated phages) in their genomes. Induction of endogenous prophages can lead to transduction of host DNA, and often leads to mobilization of other resident MGEs^4,5^. The common *S. aureus* lab strain NCTC 8325 has three prophages: ϕ11, ϕ12, and ϕ13^6,7^.

All tailed bacteriophages (order *Caudovirales*) package their genomes into preformed procapsids made from capsid protein (CP), with a ring-like portal protein (PP) forming a unique vertex at one end of the capsid^8,9^. Sometimes minor capsid proteins are incorporated. Capsid assembly typically requires a scaffolding protein (SP)^10^; however, in the HK97-like group of phages, the scaffolding protein is not separate, but constitutes an N-terminal extension—the delta (δ) domain—of CP^11^. DNA packaging requires the terminase complex, consisting of a small (TerS) and a large (TerL) subunit, where TerS recognizes the DNA to be packaged, while TerL carries out the packaging reaction in an ATP-dependent manner^12^. In headful packaging phages, like *S. aureus* phages ϕ11 and its close relative 80α, TerS recognizes *pac* sites in linear genomic concatemers, whereupon packaging proceeds until the capsid is full, typically after packaging ≈110% of the genome. The nuclease activity of TerL then cleaves the genome, leading to a circularly permuted, terminally redundant linear DNA molecule in each phage particle. In contrast, *cos* site phages, like *S. aureus* phage ϕ12, package their genomes in exact unit lengths^12^. In this case, the terminase recognizes and cleaves at a specific *cos* sequence in the genome concatemers, and packaging proceeds until the next *cos* site reaches the terminase. As long as the capsid is also full, the DNA is cleaved at the *cos* site, resulting in exact units of DNA with sticky ends being packaged into each phage particle.

Some MGEs, known as the phage-inducible chromosomal islands (PICIs)^13^, take advantage of the packaging machinery of specific “helper” phages to have their genomes packaged into phage particles at high frequency, a process that we dubbed “molecular piracy”^14,15^. The *S. aureus* pathogenicity islands (SaPIs) are the prototypical members of the PICI family and are commonly found in staphylococcal genomes^16,17^. SaPIs encode factors such as superantigen toxins that increase the virulence of the host. One group of SaPIs that includes SaPI1 and SaPIbov1 is mobilized by headful packaging phages such as ϕ11 and 80α^17^. These *pac* type SaPIs feature a “phage exploitation module”^13^, which includes a *terS* gene encoding a SaPI-specific TerS that recognizes a unique *pac* site in the SaPI genome^18^. This operon also includes *cpmA* and *cpmB* that encode two proteins (CpmA and CpmB) that redirect the capsid assembly pathway of their helper phage from large (*T* = 7) to small (*T* = 4) capsids^19,20^.

A distinct group of SaPIs that includes SaPIbov4 and SaPIbov5 lacks the classical phage exploitation module and does not encode CpmA, CpmB, or TerS^21–23^. These SaPIs encode a distinct phage interference gene cluster, comprising open reading frames (ORF) 8–12 in SaPIbov5^23^. They also encode virulence factors such as the von Willebrand factor binding protein (*vwb*) that increase invasiveness in their bovine hosts^21^. While these SaPIs can be mobilized by phages 80α and ϕ11, they do not change the size of these phages’ capsids^22^. However, we showed that SaPIbov5 is also mobilized by ϕ12, a *cos* site packaging phage with a prolate capsid, and that SaPIbov5 contains a ϕ12 *cos* site in its genome^23^. ϕ12 has an HK97-like capsid cluster that lacks a separate SP gene and encodes a herpesvirus-like protease and a CP (gp33, product of ORF33, 402 residues), with an N-terminal δ domain^24^. We showed that the presence of SaPIbov5 dramatically alters the size and shape of the capsids produced by ϕ12 from large and prolate to small and isometric, thus allowing for the packaging of the smaller SaPIbov5 genomes^23^. This size redirection was found to be dependent on the SaPIbov5-encoded protein Ccm (ORF11, 355 residues), which was also predicted to have a classical HK97-like fold with an N-terminal δ domain^23^. Presumably, *cos* type SaPIs acquired this protein horizontally from phage genomes, and evolved the ability to use it to hijack their helpers’ assembly pathways.

Here, we show that Ccm is incorporated into capsids formed in the presence of SaPIbov5, and that Ccm is the only SaPIbov5-encoded protein required to redirect capsid assembly. We also show that both CP and Ccm are cleaved during assembly to remove their δ domains. We have carried out cryo-electron microscopy (cryo-EM) and 3D reconstruction of both the prolate ϕ12 procapsids and the small, isometric SaPIbov5 procapsids. Our structures show that the ϕ12 procapsid has icosahedral end caps with *T*_end_ = 4 architecture and a *T*_mid_ = 14 cylindrical midsection, whereas SaPIbov5 capsids are icosahedral with *T* = 4 architecture. We built atomic models for CP and Ccm in the SaPIbov5 procapsid, and show that pentamers are made of Ccm, while hexamers are made of CP. Our results suggest a model for size redirection in which preferential incorporation of Ccm pentamers prevents the cylindrical midsection from forming by imposing a constant curvature on the capsid shell assembly. PICIs and other pirate elements have evolved diverse mechanisms to redirect the assembly of their phage helpers in order to promote their own replication^15^. Here, we have described an example of size redirection of a prolate capsid where the pirate element uses a phage capsid protein homolog to alter the size of its helper.

## Results

### Structure of the prolate ϕ12 procapsid

**Fig. 1:**
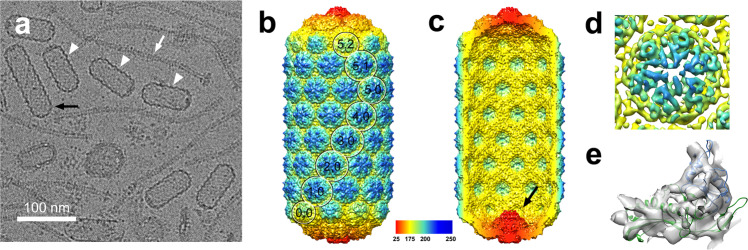
Reconstruction of the ϕ12 procapsid. **a** Representative cryo-electron micrograph of ϕ12 procapsids (white arrowheads). The white arrow indicates a phage tail; the black arrow points to a capsid with abnormal length. Scale bar = 100 nm. **b** Isosurface representation of the C5 symmetrical ϕ12 procapsid reconstruction, rendered at four standard deviations above the mean (4σ), and colored radially from the central fivefold symmetry axis according to the color bar. The *T*_mid_ = 14 lattice (*h*,*k*) is indicated. **c** Cutaway view showing the inside of the ϕ12 procapsid. The portal is visible at the bottom vertex (black arrow). **d** Close-up view of the density corresponding to one of the central midsection hexamers (5σ). **e** Ribbon diagram of HK97 gp5 (PDB ID: 3E8K) with the A- and P-domains (blue and green, respectively) individually rigid-body fitted into one subunit in the hexamer density.

ϕ12 procapsids were produced by mitomycin C induction of the *S. aureus* lysogen JP10942, which contains a ϕ12 *terS*am prophage with an amber stop codon in the *terS* gene (Supplementary Table 1). This mutation renders the phage unable to package DNA, leading to the accumulation of procapsids, which were purified by PEG precipitation and CsCl gradients. The ϕ12 procapsids were imaged by cryo-EM, which showed oblong cylindrical particles, about 40 nm wide and 82 nm long, with rounded end caps and the characteristic thick-walled, serrated appearance of phage procapsids (Fig. 1a). Many tails, which tend to co-purify with the procapsids, were also present, but very few shorter or longer capsids were observed. A dataset of 16,924 procapsid particles was subjected to 3D reconstruction in RELION 3, with the application of C5 symmetry, yielding a density map at 8.2 Å resolution (FSC = 0.143; Supplementary Fig. 1a and Supplementary Table 2).

The ϕ12 procapsid reconstruction shows the clustering of capsid protein into pentamers and hexamers, with two hemispherical end caps and a cylindrical midsection (Fig. 1b). The end caps have *T*_end_ = 4 architecture with one hexamer located between each pentamer. In the end cap at the bottom of the capsid (where the tail is attached), one pentamer is replaced by the dodecahedral portal (Fig. 1c). The portal complex forms a cone-shaped density with most of the mass inside the shell, similar to portals from other phages. Due to the applied C5 symmetry, the density for the dodecameric portal was smeared out and could not be resolved as individual proteins.

The cylindrical midsection is comprised of six rings of ten hexamers, each rotated by 18° relative to each other, forming a helix with rise 96 Å and twist 18° (Fig. 1b–d). There are seven hexamers on the shortest path between a pentamer at the bottom and a pentamer at the top, corresponding to *T*_mid_ = 14 architecture, according to the formulae *T*_end_ = *h*_1_^2^ + *h*_1_*k*_1_ + *k*_1_^2^; *T*_mid_ = *h*_1_*h*_2_ + *h*_1_*k*_2_ + *h*_2_*k*_1_ + *k*_1_*k*_2_ (*T*_mid_ is also referred to as *Q*)^25^. There are thus a total of 535 copies of CP in the capsid (30 × (*T*_end_ + *T*_mid_) – 5, assuming one pentamer has been replaced by the portal), divided into 80 hexamers and 11 pentamers. Sixty of the hexamers are in the cylindrical midsection, 40 of which are only surrounded by other hexamers and are thus in very similar environments. From the density, it was clear that the hexamers and pentamers are both made up of proteins with the expected HK97 fold, and an atomic model of HK97 gp5 (PDB ID: 3E8K) matched reasonably well upon rigid-body fitting into the ϕ12 procapsid density (Fig. 1e). However, the resolution of the map was too low for atomic modeling.

### Structure of the isometric SaPIbov5 procapsid

**Fig. 2:**
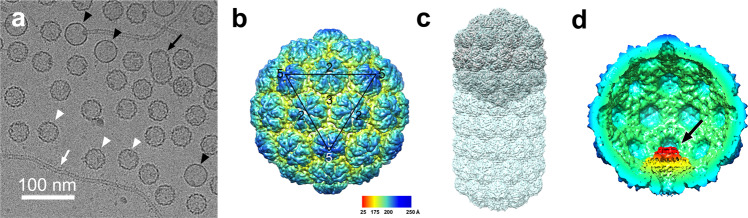
Reconstruction of the SaPIbov5 procapsid. **a** Representative cryo-electron micrograph of capsids produced by induction of strain JP12419. Procapsids and expanded capsids are indicated by white and black arrowheads, respectively. A prolate capsid is indicated by the black arrow; the white arrow points to a ϕ12 tail. Scale bar = 100 nm. **b** Isosurface representation of the icosahedral reconstruction of the SaPIbov5 (JP12419) procapsid, colored radially from the center according to the color bar. One icosahedral face is shown (black triangle), with two-, three-, and fivefold symmetry axes indicated. **c** Superposition of the SaPIbov5 (JP12419) procapsid (silver) and ϕ12 procapsid (semi-transparent blue surface) reconstructions. **d** Cutaway view of the C5 reconstruction of the SaPIbov5 (JP12419) procapsid showing the portal complex (black arrow).

SaPIbov5 procapsids were initially produced by induction of *S. aureus* lysogen JP12419, which contains ϕ12 as well as SaPIbov5*::ermC*, a version of SaPIbov5 in which the *vwb* gene had been replaced by an erythromycin resistance gene^23^ (Supplementary Table 1). This strain produces both DNA-containing virions and empty capsids and tails, which were harvested by PEG precipitation and separated on CsCl gradients. Cryo-EM of the empty capsid band showed a mixture of isometric capsids of ≈40 nm diameter with the serrated appearance of typical procapsids, and some slightly larger (≈45 nm) capsids with a smooth appearance, presumably corresponding to capsids that had expanded to their mature form (Fig. 2a). Some tails and prolate ϕ12 procapsids were also present. A dataset containing 30,870 procapsid particle images was subjected to 3D reconstruction with the application of icosahedral symmetry, reaching a resolution of 6.9 Å (FSC = 0.143; Supplementary Fig. 1b and Supplementary Table 2). The SaPIbov5 procapsids have *T* = 4 architecture with the capsid protein organized into pentamers and hexamers (Fig. 2b). Superposition of the SaPIbov5 procapsid with the ϕ12 procapsid showed that the isometric *T* = 4 capsids corresponded exactly to the *T*_end_ = 4 end caps of the prolate capsids (Fig. 2c). The density clearly showed the expected HK97-like fold of the capsid proteins, but the resolution was still too low to allow atomic modeling of the proteins into the density. 3D classification without symmetry (C1) or with C5 symmetry applied indicated that only about a third of the procapsids had portals incorporated, perhaps because procapsids that accumulate during a wild-type infection tend to be defective and unable to package DNA. A C5 reconstruction from these particles reached a resolution of 9.8 Å (Supplementary Fig. 1c and Supplementary Table 2) and clearly showed the portal complex at the unique vertex (Fig. 2d).

### Protein composition of ϕ12 and SaPIbov5 procapsids

**Fig. 3:**
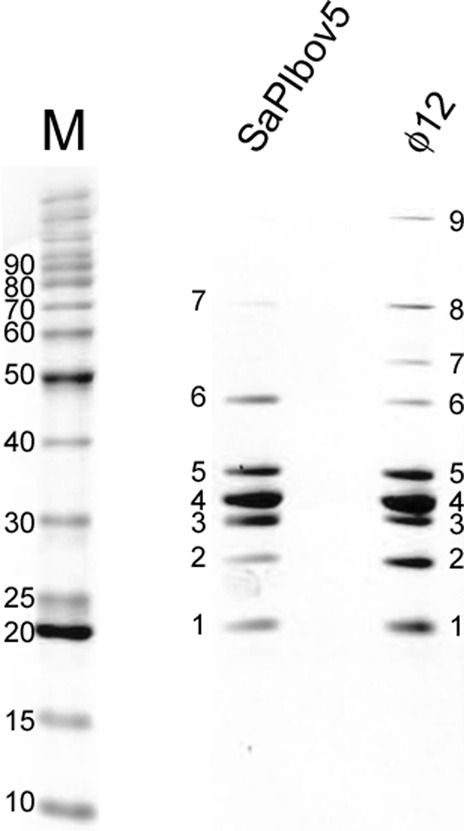
Identification of ϕ12 and SaPIbov5 proteins. SDS-PAGE separation of proteins from ϕ12 and SaPIbov5 procapsids. M, markers, molecular weights indicated (kDa). The bands that were selected for MS analysis are numbered. See Table 1.

The structural proteins present in the ϕ12 and SaPIbov5 procapsids were separated by SDS-PAGE (Fig. 3). The major bands were cut out of the gel, digested with trypsin, and subjected to peptide identification by LC-ESI-MS/MS. Apparent molecular weights were calculated relative to a second-order polynomial fit to the marker bands. Proteins identified in ϕ12 included CP (gp33), PP (gp31), the major tail protein (MTP, gp37), a minor tail protein (gp38), the tape measure protein (TMP, gp40), a tail fiber protein (Fib, gp45), and the receptor-binding protein (RBP, gp44) (Table 1). Full-length CP is 45.2 kDa, but no CP-containing band was observed corresponding to this size. Instead, CP was found in two bands with apparent molecular weights of 29.5 kDa and 32.6 kDa, suggesting that the protein had been cleaved, presumably to remove the N-terminal δ domain. Indeed, peptides corresponding to the N-terminal residues were lacking from both bands (Supplementary Fig. 2).

**Table 1:** Protein identification by MS/MS.

Band	M_r_* (kDa)	Protein ID		Calc. Mass (kDa)	Peptide Coverage (%)^†^
*ϕ12 procapsids*					
1	19.9	gp38	Minor tail protein	16.0	96
2	23.8	gp37	Major tail protein (MTP)	23.4	95
3	27.4	gp40	Tape measure protein (TMP)	226.1^§^	17^§^
4	29.5	gp33	Major capsid protein (CP)	45.2 (30.4)^¶^	60
5	32.6	gp33	Major capsid protein (CP)	45.2 (30.4)	69
6	45.0	gp31	Portal protein (PP)	40.3	80
7	54.9	gp45	Tail fiber protein (Fib)	55.4	69
8	74.7	gp44	Receptor-binding protein (RBP)	73.1	69
9	131.0	gp40	Tape measure protein (TMP)	226.1	37
*SaPIbov5 procapsids*					
1	19.9	gp38	Minor tail protein	16.0	29
2	24.0	gp37	Major tail protein (MTP)	23.4	46
3	27.4	ORF11	SaPIbov5 Ccm	40.4 (30.4)^‡^	35
4	29.5	gp33 ORF11	Major capsid protein (CP) SaPIbov5 Ccm	45.2 (30.4) 40.4 (30.4)	43 41
5	33.0	gp33	Major capsid protein (CP)	45.2 (30.4)	28
6	45.7	gp31	Portal protein (PP)	40.3	33
7	55.1	—	N.D.		

*N.D.* no phage or SaPIbov5 protein identified.

^*^Apparent molecular weights (M_r_) of the gel bands were calculated by comparison with a second-order polynomial fit of the distances of marker bands to the logarithm of their molecular weights.

^§^The tape measure protein gets cleaved into several small fragments.

^¶^The major cleaved form of CP measured by ESI-MS is 30.4 kDa.

^‡^The major cleaved form of Ccm measured by ESI-MS is 30.4 kDa.

^†^Peptide coverage was calculated in Scaffold using a 95% confidence level.

The SaPIbov5 banding pattern by SDS-PAGE was essentially identical to that of ϕ12 (Fig. 3), except that some of the higher MW bands were weak or missing, presumably due to fewer tails being present. Indeed, many of the same ϕ12 structural proteins were identified in the corresponding bands (Table 1). CP was present in bands 4 and 5, as in ϕ12. In addition, bands 3 and 4, at apparent MW of 27.4 kDa and 29.5 kDa, respectively, contained the SaPIbov5-encoded protein Ccm (ORF11), which was previously identified as responsible for capsid size redirection^23^. No other SaPIbov5-encoded proteins were detected. The apparent MW and peptide coverage of Ccm (Supplementary Fig. 2) were consistent with the removal of the N-terminal δ domain, and no band corresponding to full-length Ccm was observed. The MS results thus confirmed that Ccm is the only SaPIbov5 protein present in the small, isometric capsids formed by ϕ12 in the presence of SaPIbov5.

In order to exactly identify the cleavage sites of CP and Ccm, we subjected ϕ12 virions, produced by induction of *S. aureus* lysogen JP10435 (Supplementary Table 1), and SaPIbov5 procapsids (JP12419) to full-length mass determination by ESI-TOF-MS (Supplementary Fig. 3). In the ϕ12 virion sample, a major peak was seen at 30,374 Da, corresponding to CP cleaved between residues R128 and L129 (Table 2). The SaPIbov5 sample gave a peak at 30,373 Da, also corresponding to cleaved CP; in addition, a peak occurred at 30,431 Da, consistent with Ccm cleaved between K87 and Q88 (Supplementary Fig. 3 and Table 2). No peaks were observed at the predicted masses for full-length CP (45.2 kDa) or Ccm (40.4 kDa), consistent with the MS/MS results described above. We conclude that the δ domains constitute residues 1–128 of CP and 1–87 of Ccm and that both δ domains had already been cleaved in the procapsids that we isolated. The ϕ12 spectrum also had a peak at 23,293 Da, corresponding to the major tail protein (MTP) with the N-terminal Met removed. The identities of other, minor peaks could not be ascertained, and other structural proteins were presumably present in too small amounts to be detected by this method.

**Table 2:** Masses detected by ESI-TOF-MS.

Sample	Detected mass (Da)	Protein	Full-length mass (Da)	Sequence	Calculated mass (Da)
ϕ12	30,374.0	CP	45,234.0	129–402	30,375.2
	23,293.0	MTP	23,426.2	2–213	23,295.0
ϕ12 + SaPIbov5	30,373.0	CP	45,234.0	129–402	30,375.2
	30,431.0	Ccm	40,419.2	88–355	30,431.7

### High-resolution structure of the SaPIbov5 procapsid

Previous results indicated that *ccm* was the only gene required for redirection of ϕ12 capsid assembly, as SaPIbov5-size DNA was only observed when *ccm* was present^23^. To demonstrate directly the induction of small capsid formation by Ccm, we introduced a plasmid containing the *ccm* gene under the control of the Cd-inducible *P*_cad_ promoter (using the pCN51 vector^26^) into JP10435, yielding strain NCH04 (Supplementary Table 1). Ccm expression was induced by CdCl_2_, followed by mitomycin C induction of the prophage. After lysis, capsids were harvested by PEG precipitation and purified on preformed CsCl-step gradients.

**Fig. 4:**
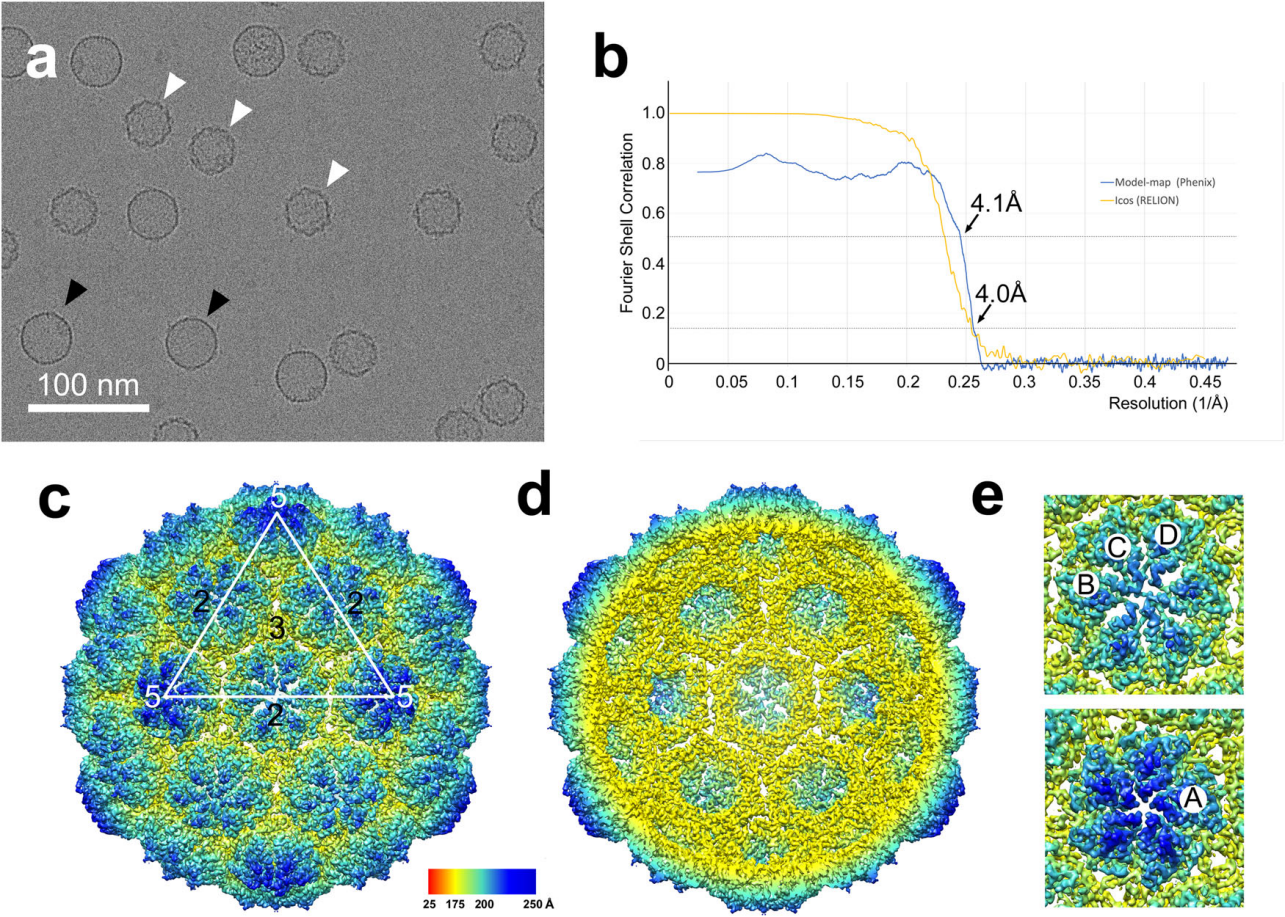
High-resolution reconstruction of the SaPIbov5 procapsid. **a** Representative cryo-electron micrograph of capsids formed upon overexpression of Ccm in the presence of ϕ12 (strain NCH04). Procapsids and expanded capsids are indicated with white and black arrowheads, respectively. Scale bar = 100 nm. **b** Fourier shell correlation (FSC) curve for the two half-maps of the SaPIbov5 procapsid reconstruction (orange). The resolution at FSC = 0.143 is indicated (4.0 Å). FSC between the map and the model is shown in blue, with the resolution at FSC = 0.5 indicated (4.1 Å). **c** Isosurface representation of the icosahedral reconstruction of the SaPIbov5 procapsids, rendered at 4σ, and colored radially according to the color bar. The white triangle represents one icosahedral face, with two-, three-, and fivefold symmetry axes indicated. **d** Cutaway view of the SaPIbov5 procapsid reconstruction. **e** Close-up of hexamer and pentamer with subunits labeled (A–D).

These capsids were imaged by cryo-EM, which showed a mixture of isometric 40 nm procapsids and 45 nm expanded capsids (Fig. 4a). An icosahedral reconstruction of the procapsids from 28,566 particle images reached a resolution of 4.0 Å (FSC = 0.143; Fig. 4b, Supplementary Fig. 1d, and Supplementary Table 2). 3D classification of the data without the application of symmetry (C1) indicated that only ≈1% of the shells had portals incorporated, and no further attempts to reconstruct in C1 or C5 were therefore made. These procapsids show the expected *T* = 4 organization with hexamer/pentamer clustering (Fig. 4c–e), and at this resolution secondary structure elements and many side chains were clearly visible, sufficient to model with confidence the proteins that make up the capsid (Supplementary Fig. 4). SaPIbov5 procapsids produced in this way were structurally identical to those produced by induction of strain JP12419, and the two reconstructions superimposed perfectly, considering the resolution difference (Supplementary Fig. 5).

**Fig. 5:**
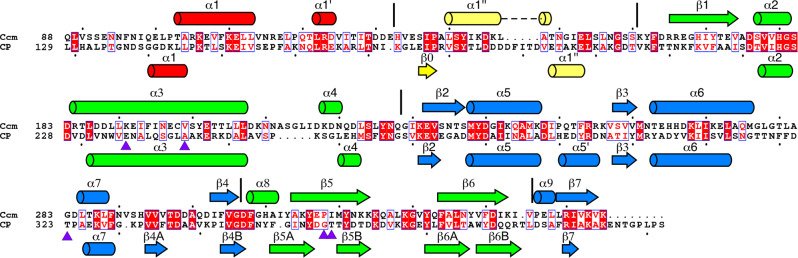
Sequence and secondary structure of Ccm and CP. Sequence alignment of Ccm and CP, generated in ESpript 3.0^46^. Secondary structure elements in Ccm and CP, determined from the high-resolution structure, are shown above and below the alignment, respectively, and colored according to domain: red, N-arm; yellow, E-loop; green, P-domain; blue, A-domain. The borders between domains are indicated by the vertical lines. Locations of CP *sir* mutations are shown as purple triangles below the sequence.

**Fig. 6:**
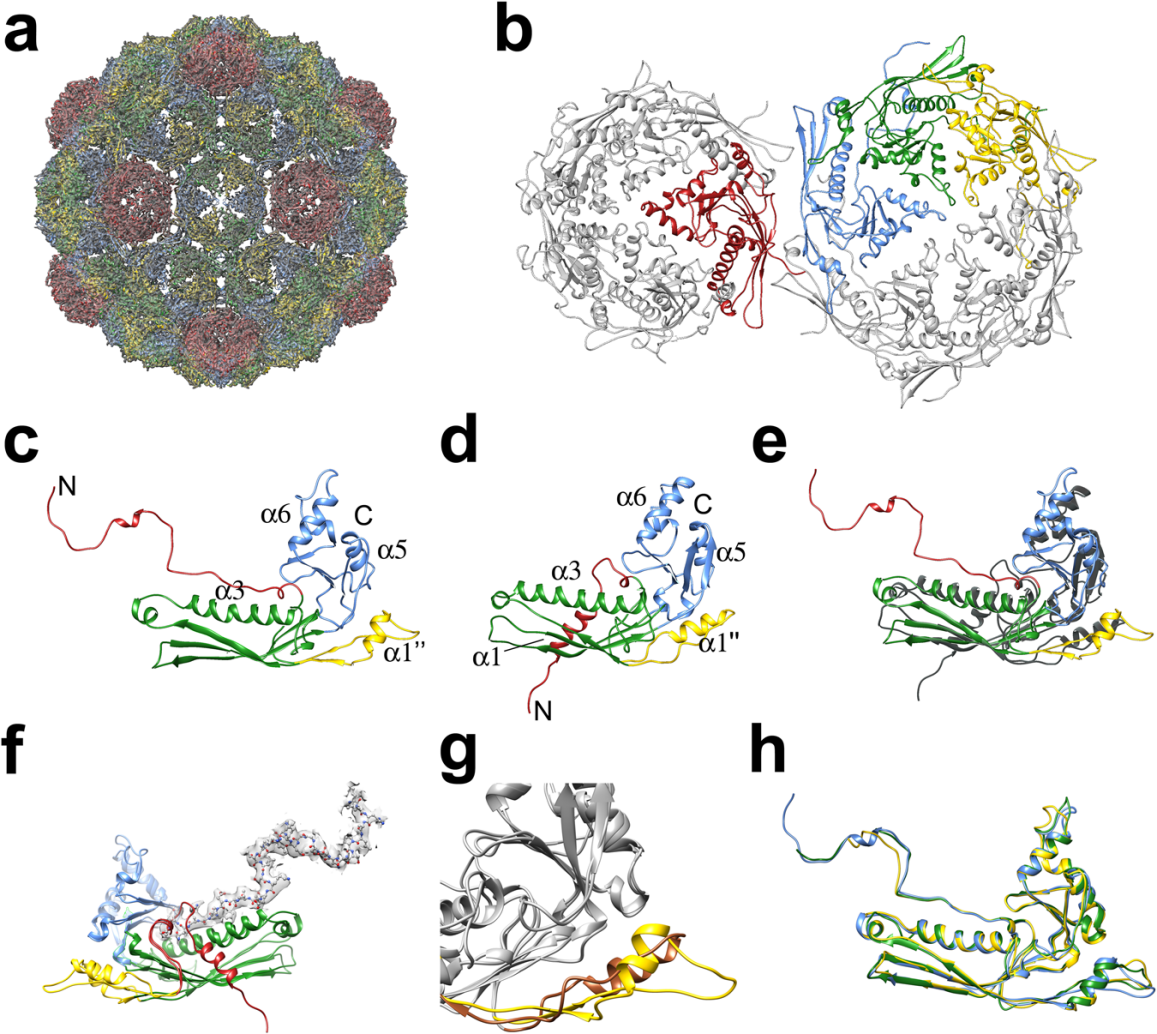
Atomic model of the SaPIbov5 procapsid. **a** Ribbon representation of the atomic model for the complete shell fitted into the density. Ccm occupies the pentamers (position A, red), while CP occupies the hexamer positions B (blue), C (green), and D (yellow). **b** Ribbon representation of one pentamer and one hexamer. The subunits corresponding to one asymmetric unit are colored as in (**a**). **c** Ribbon diagram of CP (subunit B), colored according to structural features: N-arm, red; E-loop, yellow; P-domain, green; A-domain, blue. N- and C-termini and pertinent secondary structure features are labeled. **d** Ribbon diagram of Ccm, colored and labeled as in (**c**). **e** Superposition of CP (subunit B; colored as in (**c**)) and Ccm (gray). **f** Superposition of CP and Ccm, colored as in (**c**) and viewed from the inside of the shell. The N-arm of CP is shown in ball-and-stick representation, colored according to element, with the corresponding density shown as a transparent gray surface. **g** Superposition of CP (yellow) and Ccm (brown), showing the E-loops. **h** Superposition of CP subunits B, C, and D, colored by subunit as in (**a**).

Based on the MS data, we expected the capsids to contain both CP and Ccm. A priori, the distribution of the two proteins could be random, or they could be arranged in specific positions in the shell^15^. If the distribution follows the icosahedral symmetry, the two proteins should be distinguishable in the icosahedral reconstruction. CP and Ccm have a sequence identity of 24% and were expected to be similar (Fig. 5). To initiate the modeling, I-TASSER models^27^ for both proteins were docked into all four symmetrically unique positions in the *T* = 4 shell, one in the pentamer (subunit A) and three in the hexamer (subunits B–D) (Fig. 4e). Based on the MS results, the δ domains were omitted, so that the Ccm sequence starts on residue 88, while CP starts on residue 129. Throughout the sequence, key residues could be identified that were distinctly different between CP and Ccm. For example, residues F320–F321 in CP correspond to T280–L281 in Ccm, and W234 in CP is L189 in Ccm (Fig. 5 and Supplementary Fig. 4e–g). These differences allowed us to distinguish the two proteins and identify the best matching protein in each position (A–D) in the density. Cycles of manual model building and real-space refinement demonstrated conclusively that the pentamers (subunit A) were made of Ccm while the hexamers (subunits B–D) were composed of CP (Fig. 6a, b and Supplementary Fig. 4). The final model matched the density with a model-to-map resolution of 4.1 Å (FSC = 0.5; Fig. 4b and Supplementary Table 2).

Both proteins follow the classical HK97-like fold with an N-arm, an E-loop, a P-domain that includes a long “spine helix” (α3), and an A-domain (Figs. 5 and 6b–d). The superposition of the two atomic models revealed the similarities and differences between the two proteins (Fig. 6e). Most of the two proteins are quite similar and about half of the residues in CP and Ccm could be superimposed with a root mean square deviation (RMSD) of ≈1.2 Å (Supplementary Table 3). When all matching pairs of residues were considered, however, the RMSD increased to ≥11 Å (Supplementary Table 3). One striking difference is the exceptionally long, extended N-arm in CP, especially in subunit B, where the chain could be traced all the way from the δ domain cleavage site at residue 129 (Fig. 6c–f). The Ccm N-arm could be traced from residue 93, missing the first five residues of the sequence after the δ domain cleavage site (Fig. 6d–f). The Ccm N-arm is rotated by ≈80° relative to CP around a pivot centered on CP residue L163, and folds behind the P-domain where it points towards the interior of the shell (Fig. 6d–f). Other major differences include the E-loops; unusually, both proteins have an α-helix within their E-loops, but the location of the helix is on opposite sides of the E-loop hairpin in the two proteins (Fig. 6g). The CP protein subunits in the hexamer (B, C, and D) superimpose with RMSD ≈ 0.7 Å when only the cores of the proteins are considered and ≈1.5–1.8 Å when all matching pairs are compared (Supplementary Table 3). The main differences between CP subunits are in the N-arms and E-loops (Fig. 6h).

**Fig. 7:**
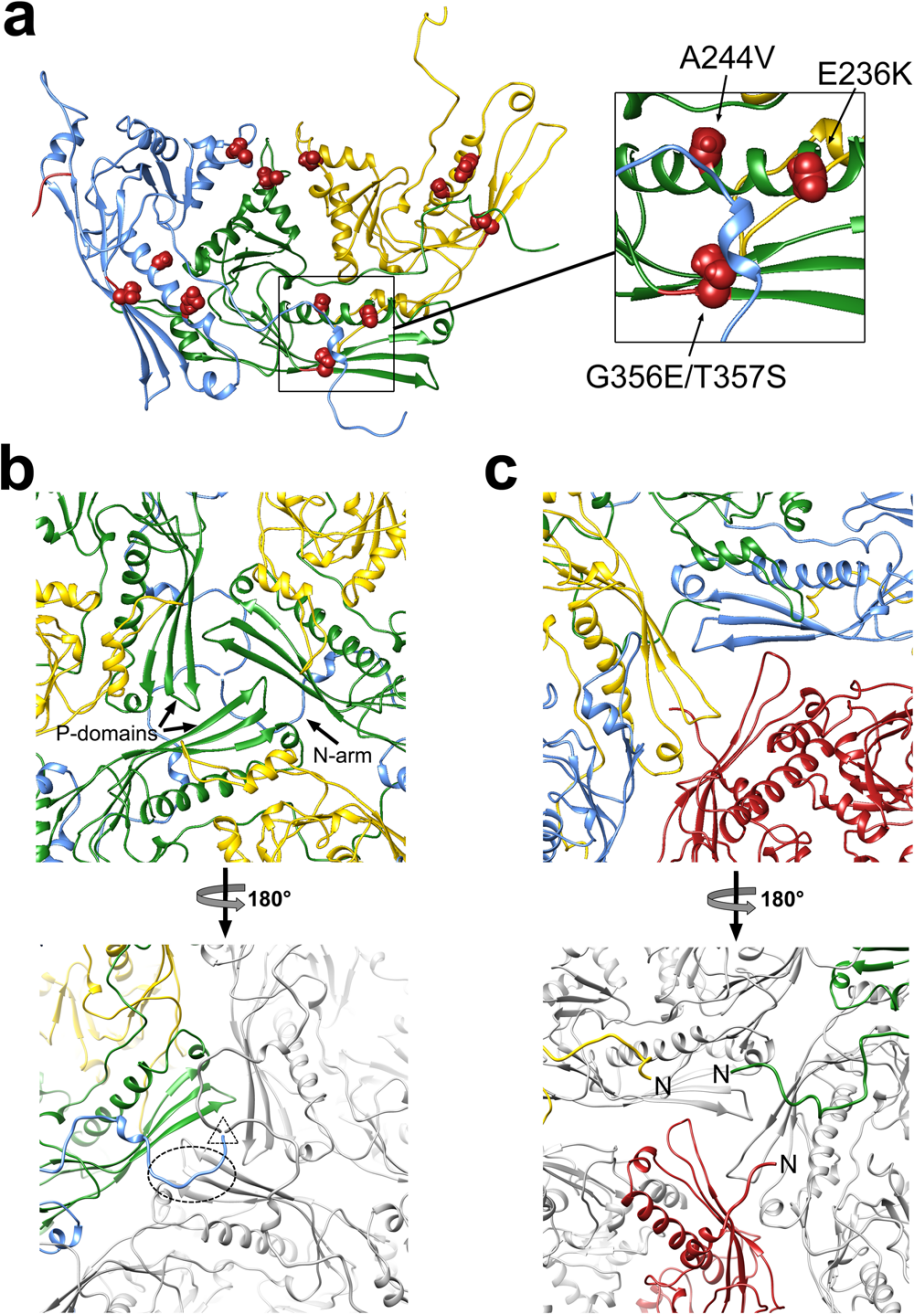
Interactions between subunits. **a** Ribbon diagram of the CP hexamer subunits B (blue), C (green), and D (yellow), viewed from the inside of the shell, showing the location of *sir* mutations (space-filling representation). The inset shows a close-up view of a B subunit N-arm (blue) interacting with the region where the *sir* residues are located in the adjacent C subunit (green). **b** Ribbon diagram showing the arrangement of subunits around the icosahedral threefold axis. The top panel is viewed from the outside of the shell, while the bottom panel is viewed from the inside. The P-domains from three C subunits (green) form a trivalent interaction. The N-arms of the B subunits (blue) reach across to interact with the C subunit in the adjacent capsomer (dashed oval outline) and come together in a triskelion (dashed triangle) at the threefold axis. **c** Ribbon diagram showing the subunits around the quasi-threefold axis between a pentamer and two hexamers, viewed from the outside (top) and from the inside (bottom). P-domains from the A (red), B (blue), and D (yellow) subunit form a trivalent interaction, but the N-arms do not form a triskelion.

Since pentamers are made up entirely of Ccm and hexamers are made up entirely of CP, intra-capsomeric interactions are only made between proteins of the same type. The A-domains of both proteins have a wedge-like shape that packs tightly around the center of the capsomers where they form interactions with their neighbors (Fig. 6a–b and Supplementary Table 4). Other intra-capsomeric interactions are formed by the E-loops, which overlap with the P-domain spine helix (α3) of the adjacent subunit on the outside of the shell (Figs. 6b and 7a and Supplementary Table 4). Unusually, the N-arm of CP, but not Ccm, stretches along the inside of the shell where it makes extensive contacts with the P-domain of the adjacent subunit (Fig. 7a and Supplementary Table 4).

In contrast, intercapsomeric interactions are sparse and mostly mediated through the P-domains, which interact around the icosahedral threefold axes (formed by three C subunits) and the quasi-threefold axes formed by A, B, and D subunits between two hexamers and a pentamer (Fig. 7b, c and Supplementary Table 4). At the icosahedral threefold axis, the N-arms from three B subunits come together to form a triskelion-like structure (Fig. 7b). The proximity of the CP N-termini at the threefold axes suggests that the δ domains may be involved in bringing the hexamers together during assembly. Because of the different orientation of the N-arm in Ccm, there is no triskelion structure formed by the N-arms at the quasi-threefold position between two hexamers and a pentamer (Fig. 7c).

## Discussion

We have determined structures of the prolate *S. aureus* phage ϕ12 procapsids and of the isometric SaPIbov5 procapsids that are formed in the presence of Ccm. Our results show that the prolate ϕ12 procapsid has *T*_end_ = 4 end caps and a *T*_mid_ = 14 cylindrical midsection, whereas the isometric SaPIbov5 procapsids have *T* = 4 architecture, suggesting that size redirection involves preventing the formation of the cylindrical midsection. Furthermore, we showed that Ccm replaces CP at the pentamers in the isometric procapsids. The introduction of Ccm pentamers is therefore responsible for shifting the assembly pathway towards small, isometric capsids, a mechanism for capsid size redirection that has not previously been described.

**Fig. 8:**
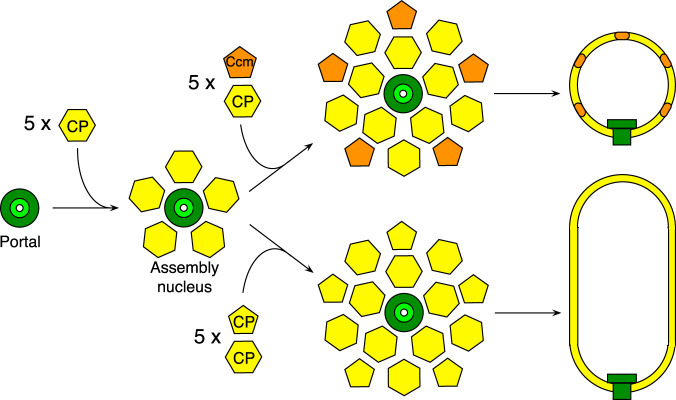
Schematic diagram of the Ccm-induced size redirection. The initial step is the formation of an assembly nucleus consisting of the portal (green) and five CP hexamers (yellow). If Ccm pentamers (orange) and CP hexamers are added to this nucleus, isometric capsids are formed; if both pentamers and hexamers of CP are used, prolate capsids result. Note that this should not be taken to imply that the capsid necessarily assembles through the addition of preformed pentamers and hexamers.

It is generally assumed that tailed phage capsid assembly starts with the portal as a nucleus. The first step in the assembly of both isometric SaPIbov5 and prolate ϕ12 capsids would therefore be the formation of a complex consisting of the portal and five CP hexamers (Fig. 8). The next step constitutes a critical decision point: incorporation of Ccm pentamers around this nucleus drives the assembly towards isometric capsids, whereas incorporation of CP pentamers leads to the formation of prolate capsids (Fig. 8).

How does the introduction of Ccm pentamers change the course of assembly from prolate to isometric? We previously noted that since isometric capsids have the highest possible ratio of pentamers to hexamers (12:20 in the case of *T* = 4 capsids vs. 12:80 for the prolate ϕ12 procapsid), simply the introduction of more pentamers could skew the distribution towards isometric capsids^15^. Capsids of intermediate size are rarely observed, however, suggesting that size redirection is a strictly either/or choice. Since CP is also capable of forming pentamers, Ccm would need to be preferentially incorporated to drive the assembly towards isometric capsids, for example by being more energetically favored than CP in the pentameric positions.

Assembly of an isometric capsid—which has a constant curvature—would seem to be a simpler case than a prolate capsid, for which the curvature varies across the lattice. How do prolate capsids regulate this transition from hemisphere to the cylinder and back again during assembly? What determines the length of the capsid? The assembly process is highly precise: ϕ12 capsids of abnormal length were exceedingly rare. The *cos* packaging mechanism requires capsids to be filled with exact multiples of the genome (typically one) from the linear concatemeric substrate. Capsids of aberrant size would likely be unable to be packaged with genomes of the correct length and are thus strongly selected against. In most phage systems, the scaffolding protein is assumed to be involved in the regulation of capsid size and shape^28,29^. However, like phage HK97, ϕ12 does not encode a separate SP. Instead, size determination is likely to involve the δ domains, perhaps by some kind of “cumulated strain” mechanism^30,31^. The portal is also likely to play a role in this process. It is notable that the isometric SaPIbov5 procapsids could form in the absence of PP, but that the prolate ϕ12 procapsids always contained portals.

Ccm might prevent this hemisphere-to-cylinder transition simply by having a more limited range of conformational plasticity. We considered whether Ccm pentamers might have a higher intrinsic curvature than CP pentamers, but our measurements showed that there are only subtle differences in the dihedral angles formed between a pentamer and the surrounding hexamers in the two shells (Supplementary Fig. 6). Instead, the change from prolate to isometric is likely to be determined through specific interactions between Ccm and adjacent CP proteins. The observed interactions between pentamers and hexamers in the SaPIbov5 procapsid are sparse (Supplementary Table 4). We suspect that the δ domains are important in this process.

Ccm alone is insufficient to form complete capsids. We previously showed that a plasmid expressing Ccm was incapable of complementing a ϕ12 ΔCP phage^23^. Furthermore, no capsids of any kind were formed when Ccm was expressed in the absence of ϕ12. This suggests that Ccm is incapable of forming the intercapsomeric contacts needed to complete capsid assembly. The formation of a closed shell requires the creation of trivalent interactions between capsomers. In the SaPIbov5 procapsid, the N-arms from three CP subunits connect in a triskelion at the icosahedral threefold axis (Fig. 7b), suggesting that the N-terminal δ domains may be involved in bringing three hexamers together at the threefold axes. In contrast, the lack of a long, extended N-arm in Ccm would preclude the formation of such triskelia (Fig. 7c), which might explain why capsids cannot be formed from Ccm alone.

In a previous study, experimental evolution of ϕ12 in the presence of SaPIbov5 yielded mutations in CP that made the phage resistant to the size redirection effect of Ccm^23^. These mutations were located in various places throughout the CP sequence (Fig. 5 and Supplementary Table 5). Two of the mutations (E236K and A244V) change the CP residue to that found in the equivalent position in Ccm, while one (T323P) changes a Thr to a Pro, where Ccm has a Gly in the same position. The last two (G356E/T357S) do not change the CP sequence to something resembling the Ccm sequence. Four of these mutations are located in a patch in the P-domain that includes the spine helix (α3) and the P-domain β-sheet, while T323P is located in a loop (the A loop) at the top of the A-domain (Figs. 5 and 7a).

We refer to these as size responsiveness (*sir*) mutations, by analogy with mutants in *Escherichia coli* bacteriophage P2 that made the P2 capsid resistant to the size redirection by the pirate element P4^32^. Structural analysis showed that these mutations in P2 disrupted the interaction between the P4 size-determination protein Sid and the gpN capsid protein^33^. In contrast, the ϕ12 *sir* mutations are not obviously in an area of interaction between CP and Ccm. Instead, the patch of four mutations in the P-domain is in an area that interacts with the N-arm of the adjacent CP subunit in the hexamer (Fig. 7a). Mutating these residues might disrupt the interaction between CP and its neighbor’s N-arm; however, how would this lead to a failure to form isometric capsids? There are at least two possible models: (1) the mutations could affect the ability of the CP N-arms to interact with the Ccm N-arms at the quasi-threefold axes. Such interactions are not apparent in the reconstruction, but they could form at an earlier stage prior to cleavage of the δ domain; (2) the mutant CP might be better at forming pentamers, allowing it to outcompete Ccm. The T323P mutation at the top of the A-domain might allow CP to pack more tightly around the fivefold axis. In addition, the *sir* mutants in the P-domain might allow the CP N-arm to fold underneath the CP subunit, similar to that of Ccm (Fig. 6c–f). It is not known if the prolate capsids formed by ϕ12 CP*sir* in the presence of Ccm still incorporate Ccm, without undergoing size redirection, or if they lack Ccm altogether. A definite answer to these questions will have to await a higher resolution structure of wild-type and mutant ϕ12 procapsids.

Our data showed that both CP and Ccm are found in their cleaved forms in both ϕ12 and SaPIbov5 procapsids and virions. This is consistent with observations in HK97, which showed that cleavage and removal of the δ domain occur early after procapsid assembly^11^. Our assumption was that the ϕ12 protease (gp32) was responsible for cleaving Ccm as well as CP. However, there is little similarity between the two observed cleavage sites other than a positively charged residue (K or R) in the P1 position. Either the ϕ12 protease is not particularly sequence-specific, or Ccm is cleaved by a different protease. Indeed, the observation of two CP-containing bands by SDS-PAGE suggests that there is a secondary cleavage site. No protease has been identified in the SaPIbov5 genome, but Ccm could potentially be cleaved by a host protease. The involvement of host proteases in phage maturation was previously described for *S. aureus* phage 80α^34,35^. The cleavage of the δ domain could be an important checkpoint during the assembly process. In the SaPIbov5 capsid, the N-arms of the B subunits come together in a triskelion at the icosahedral threefold axis at the exact point where the δ domain cleavage site is located (Fig. 7b). This suggests that bringing together CP hexamers through trimerization of their δ domains might be a driving force for capsid assembly.

Capsid size redirection by PICIs is a common occurrence throughout the bacterial realm^36,37^. By forming small capsids, phage production is strongly suppressed, providing an evolutionary advantage to the pirate element. *pac* type SaPIs, like SaPI1, have various mechanisms for suppressing phage production^13^, but for *cos* type SaPIs like SaPIbov5, capsid size redirection appears to be the main form of suppression^23^. Indeed, ϕ12 titers were 10^6^–10^7^ times higher in the presence of a *ccm* mutant than with wild-type SaPIbov5^23^. The underlying mechanisms for size redirection in these diverse systems are highly variable, however, apparently representing cases of convergent evolution^15^. The present study has elucidated a mechanism of size redirection involving a SaPI-encoded capsid protein homolog (Ccm). Remarkably, *cos* type SaPIs appear to have acquired this capsid protein homolog horizontally from a phage genome and evolved the ability to use it as a weapon against their helpers. As more pirate-helper systems are elucidated, other mechanisms for the reassembly of phage capsids by PICIs will most likely emerge, highlighting the universal importance of these processes.

## Methods

### Production of virions and procapsids

For the production of virions and procapsids, the respective lysogens (Supplementary Table 1) were grown in CY + GL^38^ at 32 °C until the absorbance at 600 nm reached 0.5 OD, upon which 2 mg mitomycin C was added per L culture. For NCH04, 0.5 µM CdCl_2_ was added at 0.1 OD followed by the addition of mitomycin C at 0.5 OD. After 2 h of growth at 32 °C, an additional 2 mg mitomycin C were added, followed by another 2 h of growth. The cultures were then incubated overnight at 4 °C. The JP10435, JP10942, and NCH04 strains had lysed completely at this point and were clarified by centrifugation at 11,000 × *g* for 45 min. For JP12419, lysis was not complete. To increase particle yield, the pellets were resuspended in 25 ml phage buffer (50 mM Tris-HCl, pH 7.8, 100 mM NaCl, 1 mM MgSO_4_, 4 mM CaCl_2_) with 5 µg lysostaphin, followed by three cycles of freeze/thaw with a final incubation at 37 °C for 1 h. The resulting lysate was centrifuged at 3600 × *g* for 10 min and the supernatant was combined with the previously clarified lysate.

The clarified lysates were made up to 10% PEG 6000 or PEG 8000 and 1.0 M NaCl and incubated overnight at 4 °C. The solutions were centrifuged at 11,000 × *g*, 45 min, and the supernatants were discarded. Pellets of JP10435, JP10942, and JP12419 were left to resuspend in phage buffer overnight at 4 °C with gentle shaking. The resuspended pellets were made up to 1.5 g/ml (JP10435, JP10942) or 1.4 g/ml (JP12419) CsCl, and centrifuged for 24 h in a Beckman NVT 90 rotor at 340,000 × *g*. The NCH04 PEG pellets were resuspended by pipetting, followed by the addition of equal volume chloroform and centrifugation at 3000 × *g* for 10 min. The aqueous phase was collected and layered on a CsCl-step gradient with densities of 1.3, 1.5, and 1.7 g/ml and centrifuged for 4 h in a SW41 rotor at 140,000 × *g*. Visible bands were removed from the tubes and dialyzed in phage dialysis buffer (20 mM Tris-HCl pH 7.8, 50 mM NaCl, 1 mM MgSO_4_, and 4 mM CaCl_2_. The SaPIbov5 procapsid band from JP12419 was further purified by centrifugation on 10-40% sucrose gradients in phage buffer for 2 h in a SW41 rotor at 118,000 × *g*. The band corresponding to procapsids was diluted 3× in phage buffer, pelleted by centrifugation at 185,000 × *g* in a Beckman 70Ti rotor, and resuspended in phage dialysis buffer.

### Mass spectrometry

For protein identification by MS, ϕ12, and SaPIbov5 procapsids were purified from strains JP10435 and JP12419, respectively, by PEG precipitation, chloroform extraction, and CsCl-gradient centrifugation. Negative stain EM confirmed that the preparations contained predominantly procapsids and tails. The samples were separated by SDS-PAGE on a 10% Bis-Tris gel and stained with colloidal Coomassie blue. The major visible bands were excised from the gel, digested with trypsin, and analyzed by LC-ESI-MS/MS, using a Thermo Orbitrap Velos Pro spectrometer. Masses were compared against a database consisting of *S. aureus* proteins, including ϕ12 and SaPIbov5. For determination of the CP and Ccm cleavage sites, equal volumes of 6 M guanidine HCl were added to samples of ϕ12 virions and SaPIbov5 empty capsids. Formic acid was added to a final concentration of 0.1% before loading on a C8 reverse-phase column. The proteins were then eluted with an acetonitrile gradient into a Waters Synapt ESI-TOF-MS instrument.

### Electron microscopy

Cryo-EM samples were prepared as previously described^20,39^. Briefly, 3 µl of sample in phage dialysis buffer were placed on glow-discharged nickel Quantifoil R2/2 grids, and vitrified using an FEI Vitrobot Mark IV. Grids were screened on an FEI Tecnai F20 microscope equipped with a Gatan K2 detector. For high-resolution data collection, the grids were sent to the SouthEastern Consortium for Microscopy of MacroMolecular Machines (SECM4) at Florida State University. A total of 2672 cryo-EM images of ϕ12 procapsids (JP10942) and 3211 images of SaPIbov5 procapsids (JP12419) were collected using Leginon 3.3 at 300 kV on a Titan Krios electron microscope equipped with a Direct Electron DE-64 detector operated in integrating mode, with 36 frames per movie and a total dose of ≈60 e^–^/Å^2^, and a pixel size of 1.01 Å (Supplementary Table 2). For the SaPIbov5 procapsids produced by Ccm overexpression (NCH04), 2570 images were collected with Leginon 3.3 on the SECM4 Titan Krios microscope using a Gatan K3 detector mounted post-GIF, with 76 frames per movie and a total dose of 60 e^–^/Å^2^, and a pixel size of 1.1 Å (Supplementary Table 2). Frame alignment with dose weighting was done with MotionCor2^40^.

### Three-dimensional reconstruction and model building

Data processing was done primarily using RELION 3.0.8^41,42^. CTF correction was done with CTFFIND4^43^ from within RELION. For the ϕ12 procapsid data, a total of 26,195 particles were picked semi-automatically using RELION and binned to 4.04 Å/pixel. After 2D and 3D classification, a subset of 16,924 particles was selected for further processing. A starting model for the 3D reconstruction was generated de novo in RELION without symmetry applied, and symmetrized to C5. High-resolution refinement was done with C5 symmetry using particles binned to 2.02 Å/pixel and a fine initial angular sampling of 1.8° to prevent the reconstruction from having pseudo-C10 symmetry, resulting in a final resolution of 8.2 Å (FSC = 0.143).

The SaPIbov5 datasets were also processed using RELION 3.0.8. For the SaPIbov5 procapsids produced by induction of strain JP12419, 62,401 particles were picked. Starting models were generated de novo. After 2D and 3D classification and selecting a subset of the best 30,870 particles, reconstruction with icosahedral symmetry yielded a reconstruction at a final resolution of 6.9 Å (binned to 2.02 Å/pixel). 3D classification with C5 symmetry showed that not all particles had a portal. The selection of the portal-containing subset (23,465 particles) yielded a reconstruction with C5 symmetry at 9.8 Å resolution.

For the SaPIbov5 procapsids produced by overexpression of Ccm (strain NCH04), a total of 53,880 particles were picked. The best 28,566 particles with the expected appearance of procapsids were selected by 2D classification and subjected to 3D refinement, using an icosahedral model generated de novo (Supplementary Fig. 7). After CTF refinement and postprocessing, the final map reached a resolution of 4.0 Å (FSC = 0.143). 3D classification without symmetry applied showed that <1% of the particles had incorporated a portal.

Initial atomic models for ϕ12 CP and SaPIbov5 Ccm were generated in I-TASSER^27^. The N-terminal δ domains (residues 1–128 for CP, 1–87 for Ccm) were left out of the models, since the MS data showed that these sequences were not present in the procapsids. The starting models were rigid-body fitted into both the hexamer and pentamer positions in the SaPIbov5 procapsid reconstruction, followed by real-space refinement in Coot 0.9.4^44^. Cycles of manual model building and real-space refinement revealed that the pentamers were made exclusively of Ccm, while the hexamers contained only CP. The model was refined in Phenix 1.18.2.^45^, using the asymmetric unit and surrounding protein chains (13 in all), resulting in a model-to-map resolution of 4.1 Å (FSC = 0.5). Refinement and validation statistics are listed in Supplementary Table 2.

### Reporting summary

Further information on research design is available in the Nature Research Reporting Summary linked to this article.

## Supplementary information

Supplementary Information

Reporting Summary

## Data Availability

The data that support this study are available from the corresponding author upon reasonable request. The three-dimensional cryo-EM density map for the SaPIbov5 procapsid reconstruction has been deposited in the Electron Microscopy Data Bank under accession number EMD-24720. Atomic coordinates for CP and Ccm in the SaPIbov5 procapsid have been deposited in the Protein Data Bank with accession number 7RWZ.
