# Supplementary material for: Shape shifter: redirection of prolate phage capsid assembly by staphylococcal pathogenicity islands
Source: Nat Commun. 2021 Nov 4;12:6408. doi: 10.1038/s41467-021-26759-x (PMC8569155; doi:10.1038/s41467-021-26759-x)
Supplement: Supplementary file 1 — Supplementary Information [file 41467_2021_26759_MOESM1_ESM.pdf]

Shape shifter: Redirection of prolate phage capsid assembly redirection by staphylococcal pathogenicity islands

N'Toia C. Hawkins[1], James L. Kizziah[1], José R. Penadés[2] and Terje Dokland[1]*

INVENTORY OF SUPPLEMENTARY INFORMATION

Supplementary Tables 1 to 5
Supplementary Figures 1 to 7
Supplementary References

SUPPLEMENTARY INFORMATION

SUPPLEMENTARY TABLES

Supplementary Table 1. Strains and plasmids used in this study

Strain	Description	Reference
RN4220	Restriction-deficient derivative of NCTC8325, cured of its prophages	[1]
JP10435	RN4220 (ϕ12)	[2]
JP12419	RN4220 (ϕ12) SaPIbov5::*ermC*	[2]
JP10942	RN4220 (ϕ12 *terS*am)	This study
NCH04	JP10435 + pJP1730	This study
**Plasmid**	**Description**	**Reference**
pCN51	Shuttle plasmid with P_{cad} promoter	[3]
pJP1730	pCN51-*ccm*	[2]

Supplementary Table 2. Reconstruction and refinement statistics

<table>
<tr><th></th><th>ϕ12 procapsids</th><th colspan="3">SaPIbov5 procapsids</th></tr>
<tr><td>Strain</td><td>JP10942</td><td colspan="2">JP12419</td><td>NCH04</td></tr>
<tr><td colspan="5"></td></tr>
<tr><td><u>Reconstruction statistics (RELION)</u></td><td></td><td colspan="2"></td><td></td></tr>
<tr><td>Microscope</td><td>Titan Krios</td><td colspan="2">Titan Krios</td><td>Titan Krios</td></tr>
<tr><td>Detector</td><td>DE-64</td><td colspan="2">DE-64</td><td>K3</td></tr>
<tr><td colspan="5"></td></tr>
<tr><td>Voltage (kV)</td><td>300</td><td colspan="2">300</td><td>300</td></tr>
<tr><td>No. Images (total)</td><td>2,672</td><td colspan="2">3,211</td><td>2,570</td></tr>
<tr><td>No. Images (used)</td><td>1,174</td><td colspan="2">1,520</td><td>2,373</td></tr>
<tr><td>Detector pixel size (Å)</td><td>1.01</td><td colspan="2">1.01</td><td>1.10</td></tr>
<tr><td>No. frames per image</td><td>36</td><td colspan="2">36</td><td>76</td></tr>
<tr><td>Total electron dose ($e^{-}/Å^{2}$)</td><td>59.0</td><td colspan="2">60.5</td><td>59.9</td></tr>
<tr><td colspan="5"></td></tr>
<tr><td>Symmetry</td><td>C5</td><td>Icos (I1)</td><td>C5</td><td>Icos (I1)</td></tr>
<tr><td>No. particles (initial)</td><td>26,195</td><td>62,401</td><td>62,401</td><td>53,880</td></tr>
<tr><td>No. particles (final)</td><td>16,924</td><td>30,870</td><td>23,465</td><td>28,566</td></tr>
<tr><td>Box size (pixels^{2})</td><td>600</td><td>300</td><td>300</td><td>512</td></tr>
<tr><td>Final pixel size (Å)</td><td>2.02</td><td>2.02</td><td>2.02</td><td>1.10</td></tr>
<tr><td colspan="5"></td></tr>
<tr><td>Resolution ($FSC_{0.143}$) (Å)</td><td>8.2</td><td>6.9</td><td>9.8</td><td>4.0</td></tr>
<tr><td colspan="5"></td></tr>
<tr><td><u>Refinement and validation statistics (Phenix):</u></td><td></td><td colspan="2"></td><td></td></tr>
<tr><td>Half-map resolution ($FSC_{0.143}$) (Å)</td><td></td><td colspan="2"></td><td>3.9</td></tr>
<tr><td>Model-to-map resolution ($FSC_{0.5}$) (Å)</td><td></td><td colspan="2"></td><td>4.1</td></tr>
<tr><td>Model to map correlation (CCmask)</td><td></td><td colspan="2"></td><td>0.85</td></tr>
<tr><td colspan="5"></td></tr>
<tr><td>Polypeptide chains (total / a.u.)</td><td></td><td colspan="2"></td><td>13/4</td></tr>
<tr><td>Total number of atoms</td><td></td><td colspan="2"></td><td>53,034</td></tr>
<tr><td colspan="5"></td></tr>
<tr><td>RMSD bonds (Å)</td><td></td><td colspan="2"></td><td>0.004</td></tr>
<tr><td>RMSD angles (°)</td><td></td><td colspan="2"></td><td>0.566</td></tr>
<tr><td colspan="5"></td></tr>
<tr><td>Molprobity score</td><td></td><td colspan="2"></td><td>1.87</td></tr>
<tr><td>Clashscore (all / a.u. only)</td><td></td><td colspan="2"></td><td>8.32/3.12</td></tr>
<tr><td colspan="5"></td></tr>
<tr><td>Ramachandran statistics:</td><td></td><td colspan="2"></td><td></td></tr>
<tr><td>Z-score</td><td></td><td colspan="2"></td><td>-1.53±0.14</td></tr>
<tr><td>Outliers (%)</td><td></td><td colspan="2"></td><td>0</td></tr>
<tr><td>Allowed (%)</td><td></td><td colspan="2"></td><td>6.45</td></tr>
<tr><td>Favored (%)</td><td></td><td colspan="2"></td><td>93.55</td></tr>
<tr><td>Rotamer outliers (%)</td><td></td><td colspan="2"></td><td>0</td></tr>
</table>

Supplementary Table 3. Root mean square deviations between CP and Ccm subunits (Cα atoms only)

Pruned RMSD		Ccm	CP		
		A	B	C	D
Ccm	A	0			
CP	B	1.15 (119)*	0		
	C	1.17 (115)	0.72 (229)	0	
	D	1.18 (116)	0.72 (209)	0.71 (221)	0
Unpruned RMSD		Ccm	CP		
		A	B	C	D
Ccm	A	0			
CP	B	11.00 (221)	0		
	C	11.59 (236)	1.51 (260)	0	
	D	11.62 (236)	1.79 (253)	1.48 (253)	0

*RMSD values in Å, with the number of equivalent Cα atom pairs compared in parentheses.

Supplementary Table 4. Interactions between subunits.

<table>
<tr><th colspan="2">Subunits§</th><th colspan="3">No. residue pairs <3.5Å¶</th><th colspan="3">No. residue pairs involving the listed structural features</th></tr>
<tr><td colspan="2">Intra-pentamer</td><td colspan="3"></td><td>N-arm</td><td>E-loop</td><td>A-domain</td></tr>
<tr><td>A</td><td>A_5</td><td colspan="3">17</td><td>4</td><td>5</td><td>4</td></tr>
<tr><td colspan="2">Intra-hexamer</td><td colspan="3"></td><td></td><td></td><td></td></tr>
<tr><td>B</td><td>C</td><td colspan="3">43</td><td>20</td><td>12</td><td>11</td></tr>
<tr><td>C</td><td>D</td><td colspan="3">37</td><td>16</td><td>14</td><td>7</td></tr>
<tr><td>D^2</td><td>B</td><td colspan="3">35</td><td>31</td><td>0</td><td>4</td></tr>
<tr><td colspan="2"></td><td colspan="3"></td><td></td><td></td><td></td></tr>
<tr><td colspan="2">Pentamer-Hexamer</td><td colspan="3"></td><td>N-arm</td><td>E-loop</td><td>P-domain</td></tr>
<tr><td>A</td><td>B</td><td colspan="3">1</td><td>0</td><td>1</td><td>0</td></tr>
<tr><td>A</td><td>D_2</td><td colspan="3">9</td><td>2</td><td>0</td><td>7</td></tr>
<tr><td colspan="2">Hexamer-hexamer</td><td colspan="3"></td><td></td><td></td><td></td></tr>
<tr><td>B</td><td>C_3</td><td colspan="3">8</td><td>8</td><td>0</td><td>0</td></tr>
<tr><td>B</td><td>D_3</td><td>7</td><td>0</td><td>0</td><td colspan="3">7</td></tr>
<tr><td>C</td><td>C_3</td><td colspan="3">5</td><td>0</td><td>0</td><td>5</td></tr>
</table>

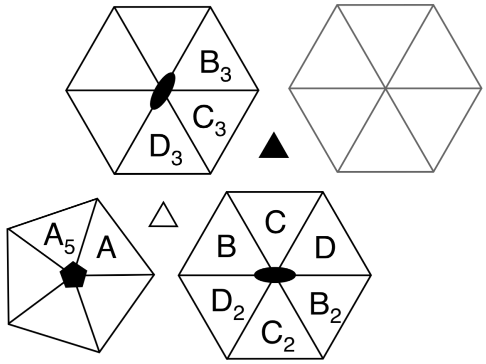

§ A subunits are made of the SaPIbov5-encoded Ccm protein, whereas B–D subunits are made of the ϕ12-encoded CP. Symmetry-related subunits are defined according to the schematic: A_5 refers to the fivefold related A subunit in the pentamer; B_2, C_2 and D_2 are the twofold related B, C and D subunits within the hexamer; B_3, C_3 and D_3 are the threefold related B, C and D subunits in the adjacent hexamer. (Subunit pairs that are not listed did not form any contacts.)

¶ Inter-atomic distances between pairs of subunits were calculated in UCSF Chimera. Two residues that formed one or more inter-atomic contacts of ≤3.5Å were counted as one residue pair.

Supplementary Table 5. ϕ12 CP size responsiveness (*sir*) mutations

CP residue	Mutation	Ccm residue	Location	Notes
E236	→ K	K	Spine helix (α3)	May affect the path of neighboring subunit's N-arm
A244	→ V	V	Spine helix (α3)	Interacts directly with N-arm of neighboring subunit
T323	→ P	G	A-loop (α6-α7)	Involved in interactions between A-domains
G356/T357	→ ES	PI	P-domain (β6)	Interacts directly with N-arm of neighboring subunit

SUPPLEMENTARY FIGURES

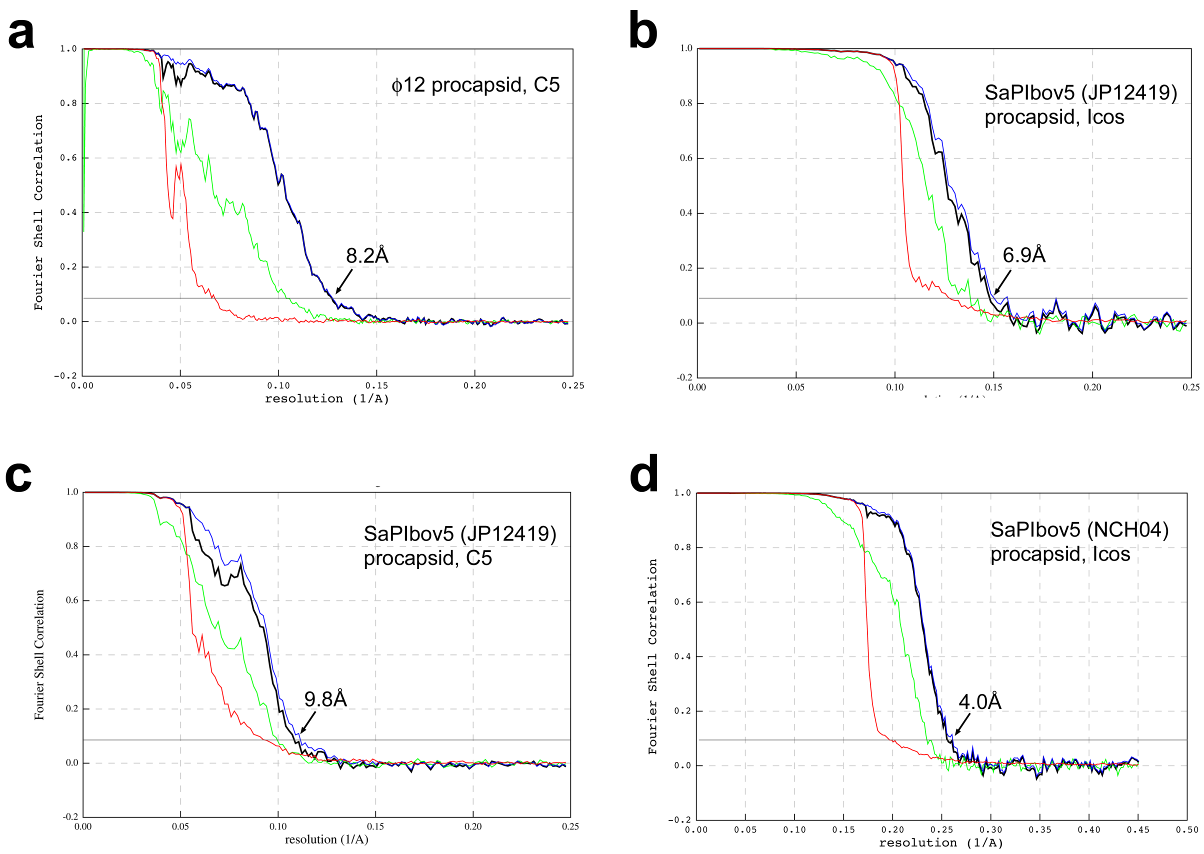

Supplementary Figure 1. FSC curves for the reconstructions. a ϕ12 procapsid (strain JP10942), C5 symmetry; **b** SaPIbov5 procapsid (strain JP12419), icosahedral; **c** SaPIbov5 procapsid (strain JP12419), C5; **d** SaPIbov5 procapsid (strain NCH04), icosahedral. All curves generated in RELION. Red curve, phase randomized; green, unmasked; blue, masked; black, corrected. The resolution at FSC=0.143 (gray line) is indicated for each curve.

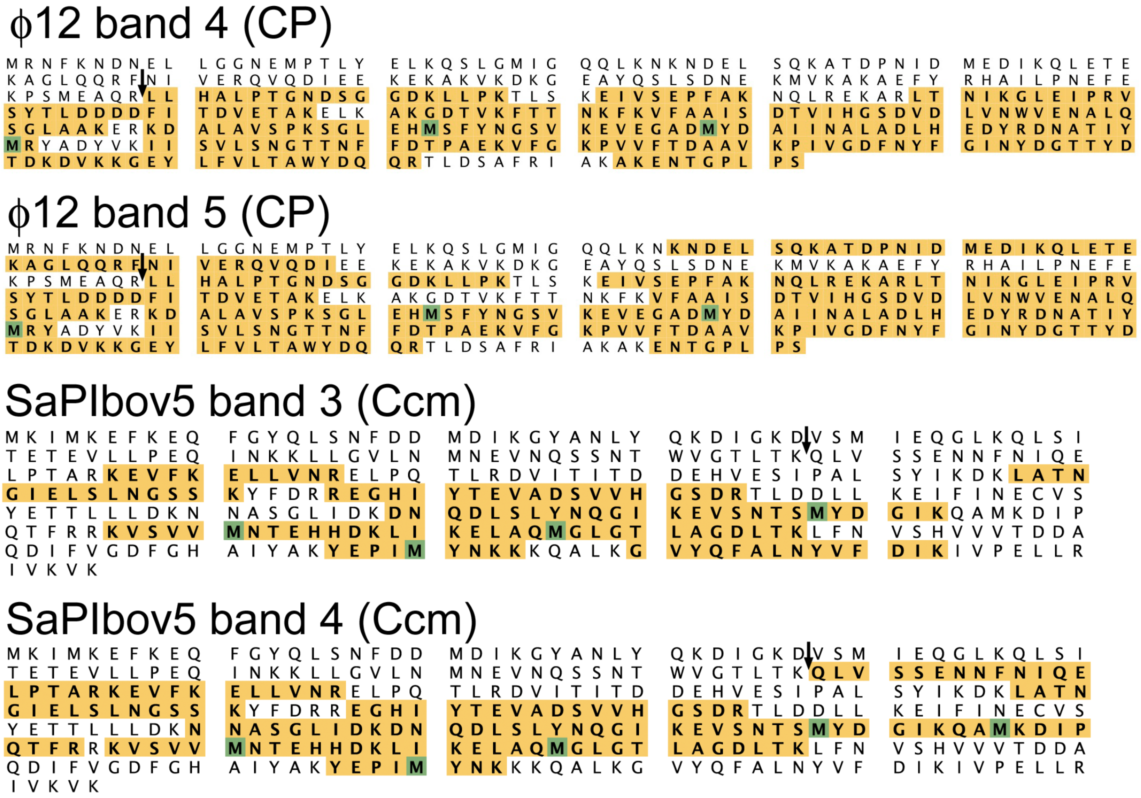

Supplementary Figure 2. Protein identification by MS. Peptide coverage (at 95% confidence level) for CP in ϕ12 bands 4 and 5 and Ccm in SaPIbov5 bands 3 and 4 (yellow highlights). The cleavage sites determined by ESI-MS are indicated by arrows. (Methionines are in green.)

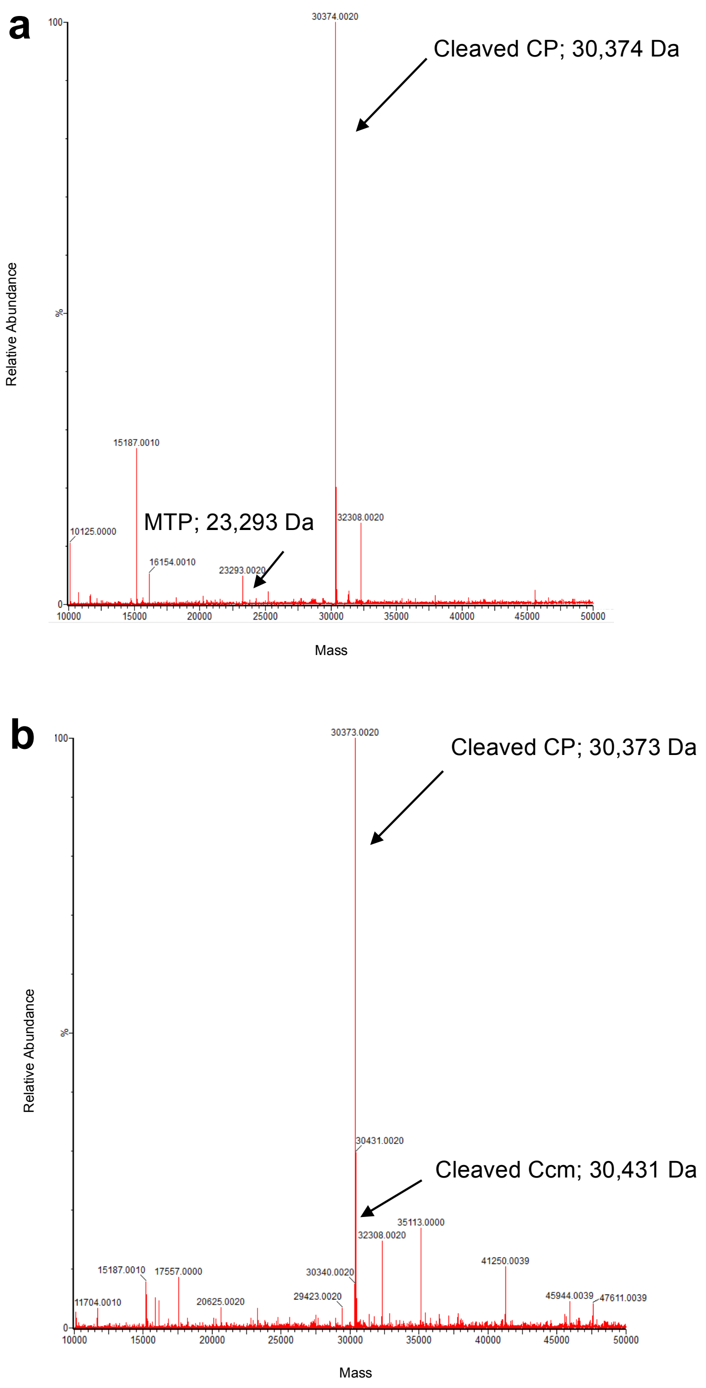

Supplementary Figure 3. Protein mass determination. Mass spectra from ESI-TOF-MS of proteins from purified ϕ12 virions **(a)** and SaPIbov5 empty capsids **(b)**. The peaks corresponding to cleaved CP, cleaved Ccm and MTP are indicated, with their measured masses shown.

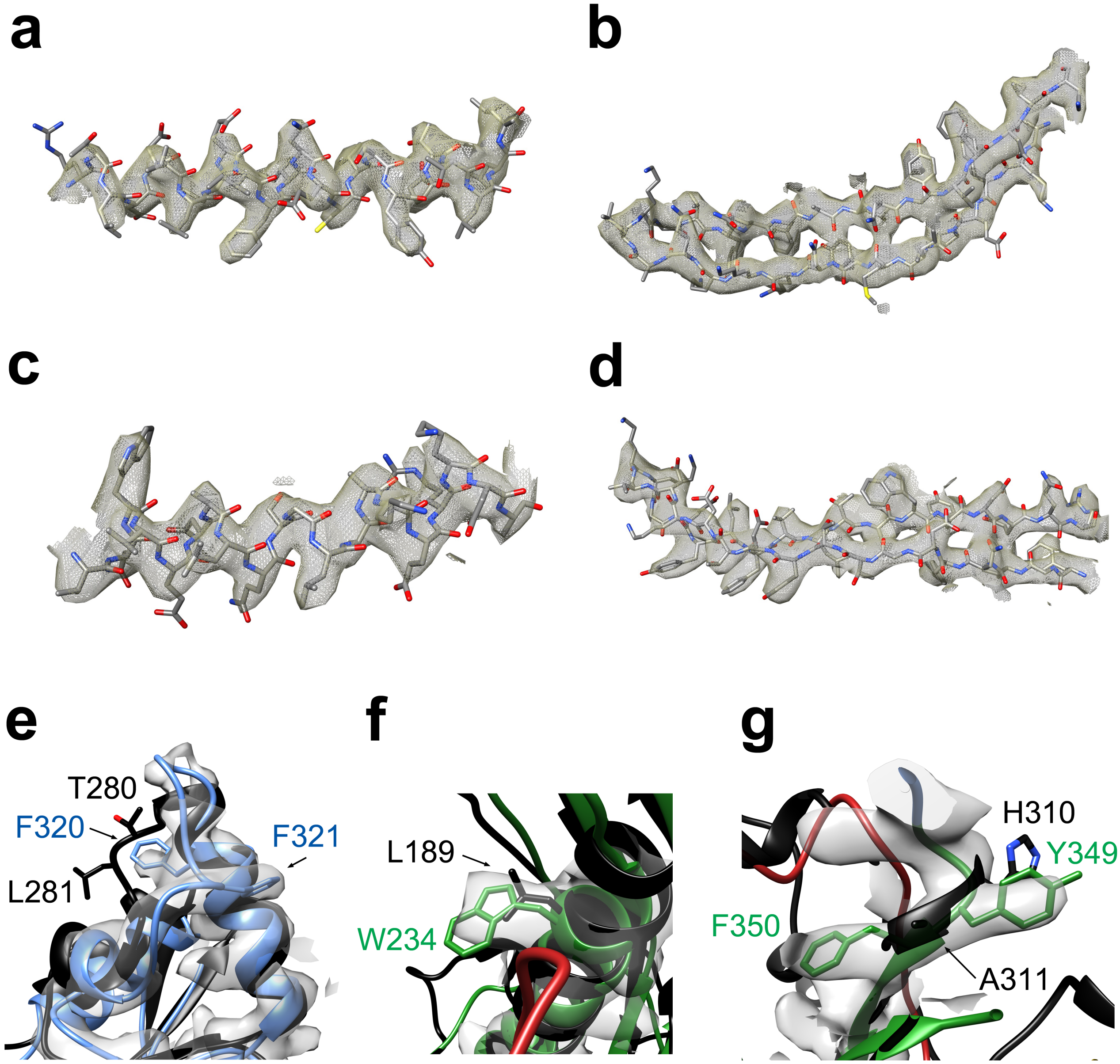

Supplementary Figure 4. Details of density from the SaPIbov5 procapsid reconstruction with the atomic model fitted in. a Spine helix (α3) of Ccm, residues R184–L207. **b** Part of P domain β sheet (β5–β6) of Ccm, residues A314–K343. **c** Spine helix (α3) of CP, residues V232–A251. **d** β5–β6 of CP, residues F349–R382. **e–g** Comparison of the CP and Ccm models. For the ribbon diagrams, CP is colored according to domain (as in Fig. 5), Ccm is shown in black. Critical residues are shown in stick representation. The density around CP is shown as a transparent isosurface: **e** F320-F321 in CP, T280-L281 in Ccm; **f** W234 in CP, L189 in Ccm; **g** Y349-F350 in CP; H310-A311 in Ccm.

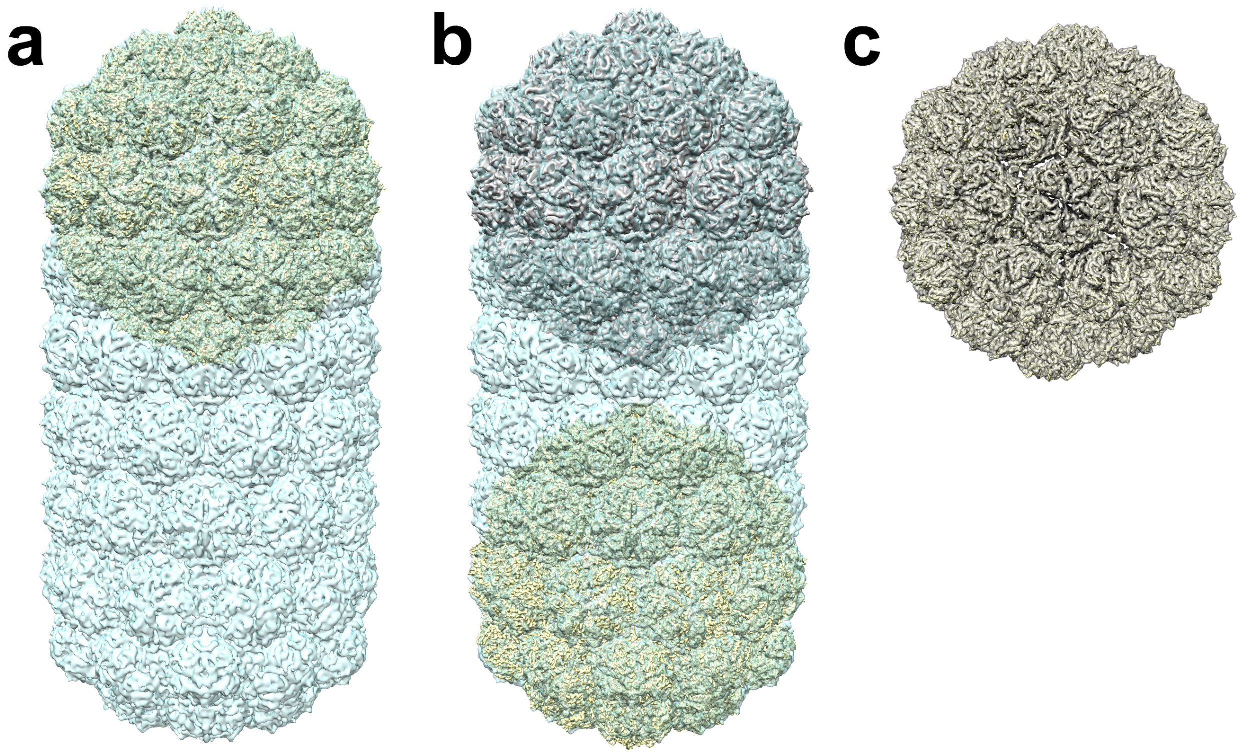

Supplementary Figure 5. Map superpositions. a Superposition of the high-resolution SaPIbov5 (NCH04) procapsid reconstruction (yellow) with the ϕ12 procapsid (transparent cyan). **b** Superposition of the JP12419 (silver) and NCH04 (yellow) reconstructions at opposite ends of the ϕ12 procapsid. **c** Superposition of the high-resolution NCH04 (yellow) and low-resolution JP12419 (silver) SaPIbov5 procapsid reconstructions.

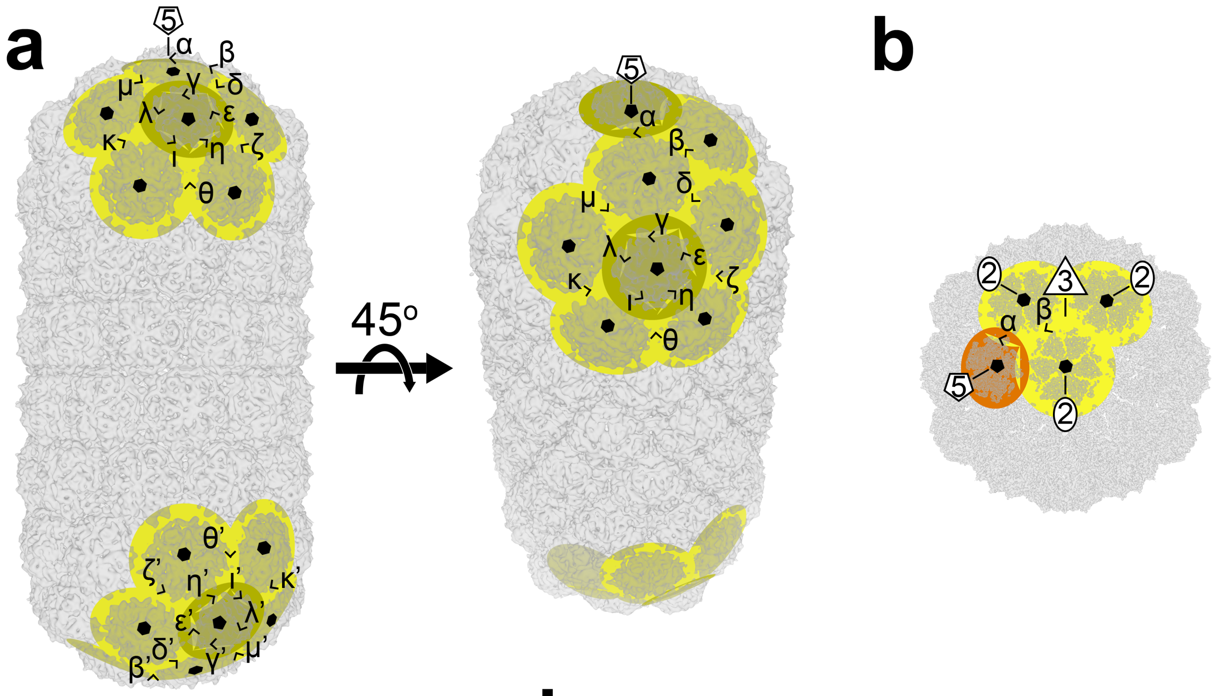

c

	φ12 procapsid					SaPIbov5	
	Top	Bottom (portal)		Δ			Δ
α	149.0				α	148.3	0.7
β	144.7	β'	145.6	-0.9	β	144.0	0.7
γ	147.6	γ'	146.7	0.9	α	148.3	-0.7
δ	142.6	δ'	142.2	0.4	β	144.0	-1.4
ε	148.2	ε'	148.6	-0.4	α	148.3	-0.1
ζ	146.9	ζ'	146.9	0.0	β	144.0	2.9
η	148.2	η'	148.6	-0.4	α	148.3	-0.1
θ	140.0	θ'	140.2	-0.2	β	144.0	-4.0
ι	148.2	ι'	148.0	0.2	α	148.3	-0.1
κ	146.9	κ'	146.8	0.1	β	144.0	2.9
λ	148.3	λ'	147.8	0.5	α	148.3	0.0
μ	142.7	μ'	142.2	0.5	β	144.0	-1.3

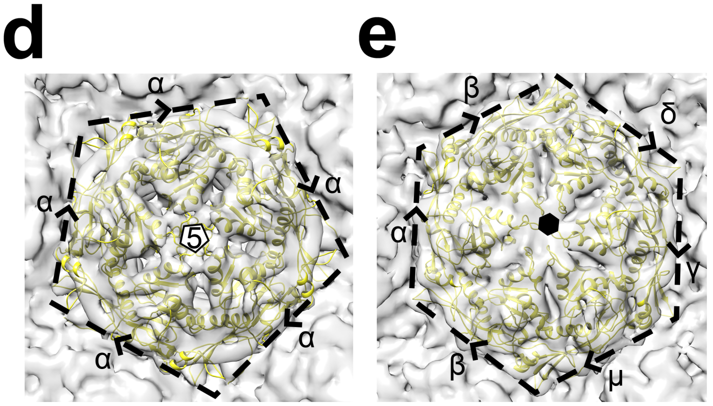

Supplementary Figure 6. Comparison of dihedral angles between capsomers in the φ12 and SaPIbov5 procapsids. The atomic model of the CP hexamer from the SaPIbov5 reconstruction and a CP pentamer model generated by replacing Ccm from SaPIbov5 with the CP sequence were rigid body fitted into the φ12 density. Dihedral angles were calculated in Chimera by drawing a plane through an invariant Cα atom in the P domains in the hexamers and pentamers. **a** The φ12 reconstruction with the hexamers and pentamers and the angles between them indicated. The fivefold axis is indicated by the open pentagon labeled '5'. The right hand panel is rotated by 45° to show the capsomers at the top of the capsid. **b** The SaPIbov5 reconstruction with the capsomers and angles indicated. Ccm is shown in orange, CP is yellow. 5-, 3- and 2-fold symmetry axes are indicated. **c** Table of dihedral angles, as defined in **a** and **b**. 180° would be completely flat (i.e. no curvature). The differences (Δ) in angles around the pentamers at the top and the pentamer at the bottom, and between corresponding anges in SaPIbov5 and φ12 are indicated. **d** Fit of a CP pentamer (yellow model) into the φ12 density at the top of the capsid. The fivefold symmetry axis is indicated, and the location of the α dihedral angle is indicated. **e** Fit of the CP hexamer (yellow model) into the φ12 density adjacent to the top pentamer. The corresponding dihedral angles (α, β, γ, δ and μ) are indicated.

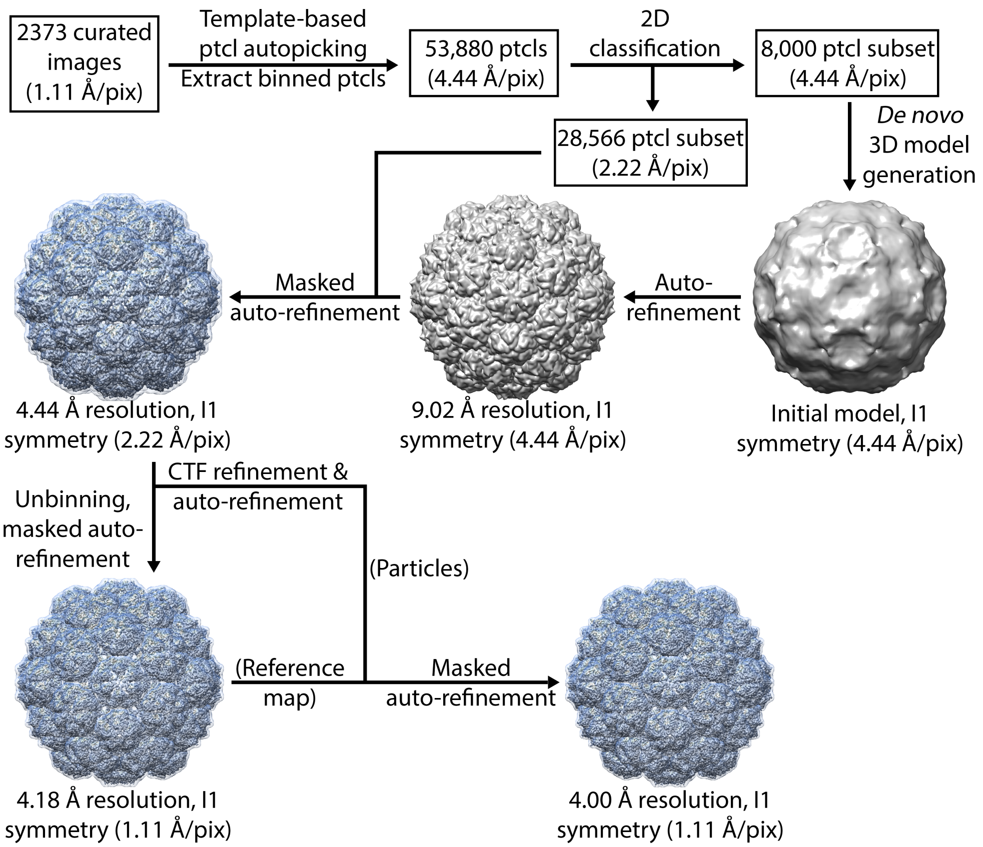

Supplementary Figure 7. Schematic workflow diagram for the high-resolution NCH04 SaPIbov5 procapsid reconstruction. Maps are in gray; masks are shown as transparent blue surfaces. The pixel size and resolution attained at each step are indicated.

SUPPLEMENTARY REFERENCES

1. Kreiswirth, B. N., Lofdahl, S., Betley, M. J., O'Reilly, M., Schlievert, P. M., Bergdoll, M. S. & Novick, R. P. (1983). The toxic shock syndrome exotoxin structural gene is not detectably transmitted by a prophage. *Nature* **305**, 709-712.
2. Carpena, N., Manning, K. A., Dokland, T., Marina, A. & Penades, J. R. (2016). Convergent evolution of pathogenicity islands in helper cos phage interference. *Phil. Trans. Roy. Soc. B* **371**, 20150505.
3. Charpentier, E., Anton, A. E., Barry, P., Alfonso, B., Fang, Y. & Novick, R. P. (2004). Novel cassette-based shuttle vector system for gram-positive bacteria. *Appl. Env. Microbiol.* **70**, 6076-6085.
