# Supplementary material for: Shape shifter: redirection of prolate phage capsid assembly by staphylococcal pathogenicity islands
Source: Nat Commun. 2021 Nov 4;12:6408. doi: 10.1038/s41467-021-26759-x (PMC8569155; doi:10.1038/s41467-021-26759-x)
Supplement: Supplementary file 2 — Reporting Summary [file 41467_2021_26759_MOESM2_ESM.pdf]

nature portfolio

Corresponding author(s): Terje Dokland

Last updated by author(s): Oct 18, 2021

Reporting Summary

Nature Portfolio wishes to improve the reproducibility of the work that we publish. This form provides structure for consistency and transparency in reporting. For further information on Nature Portfolio policies, see our Editorial Policies and the Editorial Policy Checklist.

Please do not complete any field with "not applicable" or n/a. Refer to the help text for what text to use if an item is not relevant to your study. For final submission: please carefully check your responses for accuracy; you will not be able to make changes later.

Statistics

For all statistical analyses, confirm that the following items are present in the figure legend, table legend, main text, or Methods section.

n/a	Confirmed	
☐	☒	The exact sample size (*n*) for each experimental group/condition, given as a discrete number and unit of measurement
☐	☒	A statement on whether measurements were taken from distinct samples or whether the same sample was measured repeatedly
☒	☐	The statistical test(s) used AND whether they are one- or two-sided<br>*Only common tests should be described solely by name; describe more complex techniques in the Methods section.*
☒	☐	A description of all covariates tested
☒	☐	A description of any assumptions or corrections, such as tests of normality and adjustment for multiple comparisons
☐	☒	A full description of the statistical parameters including central tendency (e.g. means) or other basic estimates (e.g. regression coefficient) AND variation (e.g. standard deviation) or associated estimates of uncertainty (e.g. confidence intervals)
☒	☐	For null hypothesis testing, the test statistic (e.g. *F*, *t*, *r*) with confidence intervals, effect sizes, degrees of freedom and *P* value noted<br>*Give P values as exact values whenever suitable.*
☒	☐	For Bayesian analysis, information on the choice of priors and Markov chain Monte Carlo settings
☒	☐	For hierarchical and complex designs, identification of the appropriate level for tests and full reporting of outcomes
☒	☐	Estimates of effect sizes (e.g. Cohen's *d*, Pearson's *r*), indicating how they were calculated

Our web collection on statistics for biologists contains articles on many of the points above.

Software and code

Policy information about availability of computer code

Data collection	Cryo-EM data were collected on a Titan Krios electron microscope equipped with a Gatan K3 direct electron detector using Leginon 3.3 software. Motion correction was done using MotionCor2.
Data analysis	Data processing was done with RELION-3.0.8. CTF correction was done with CTFFIND4 (v4.1) from within the RELION GUI. Initial atomic models were generated with the I-TASSER server (v5.1). Atomic modeling was done with Coot 0.9.4 followed by refinement in Phenix 1.18.2. The models were validated using the MolProbity server (v.4.5). Visualization was done in UCSF Chimera v1.15. Sequence alignments were done with the Clustal Omega (ClustalW) server (v1.2) and rendered in ESPript 3.0.

For manuscripts utilizing custom algorithms or software that are central to the research but not yet described in published literature, software must be made available to editors and reviewers. We strongly encourage code deposition in a community repository (e.g. GitHub). See the Nature Portfolio guidelines for submitting code & software for further information.

Data

Policy information about availability of data

All manuscripts must include a data availability statement. This statement should provide the following information, where applicable:

- Accession codes, unique identifiers, or web links for publicly available datasets
- A description of any restrictions on data availability
- For clinical datasets or third party data, please ensure that the statement adheres to our policy

The three-dimensional cryo-EM density map for the SaPIbov5 procapsid reconstruction has been deposited in the Electron Microscopy Data Bank under accession number EMD-24720 [https://www.ebi.ac.uk/emdb/EMD-24720]. Atomic coordinates for CP and Ccm in the SaPIbov5 procapsid have been deposited in the Protein

[illegible]
repository, but will be made available from the authors upon request.

Field-specific reporting

Please select the one below that is the best fit for your research. If you are not sure, read the appropriate sections before making your selection.

☒ Life sciences ☐ Behavioural & social sciences ☐ Ecological, evolutionary & environmental sciences

For a reference copy of the document with all sections, see nature.com/documents/nr-reporting-summary-flat.pdf

Life sciences study design

All studies must disclose on these points even when the disclosure is negative.

Sample size	The sample size is the number of particles used to calculate each 3D structure. The FSC resolution calculation implicitly includes the sample size in its calculation. At least 26,000 particles were used for each reconstruction, sufficient to reach the given resolution according to the FSC calculation in RELION. The icosahedral symmetry (SaPIbov5 procapsid) effectively multiplies the data set size by 60x, while the C5 symmetry (phi12 procapsid) multiplies by 5x. The final resolution achieved was not limited by the sample size, but by sample and image quality.
Data exclusions	The data were subjected to reference-free 2D classification. Classes were excluded based on semi-objective criteria, such as noise or lack of identifiable features. The final data sets included from 16,900 to 30,900 particles.
Replication	Single-particle reconstruction is based on averaging individual particle images. Replication is thus implicit in the data set and not per se required to ensure statistical robustness of the structural data. For each reconstruction, the data was split in two half data-sets, which were processed independently. The resolution was determined by the Fourier Shell Criterion between the two independent half maps. The three reconstructions shown here (phi12 procapsid, and two different SaPIbov5 procapsids) are made of the same proteins and were consistent to the given resolution and could be considered biological replicates of the same data.
Randomization	For FSC calculations, the data sets were divided randomly into two halves, which were processed independently according to the "gold standard" criterion.
Blinding	Data sets were divided randomly by software with no input from investigators.

Reporting for specific materials, systems and methods

We require information from authors about some types of materials, experimental systems and methods used in many studies. Here, indicate whether each material, system or method listed is relevant to your study. If you are not sure if a list item applies to your research, read the appropriate section before selecting a response.

Materials & experimental systems

n/a	Involved in the study	
☒	☐	Antibodies
☒	☐	Eukaryotic cell lines
☒	☐	Palaeontology and archaeology
☒	☐	Animals and other organisms
☒	☐	Human research participants
☒	☐	Clinical data
☒	☐	Dual use research of concern

Methods

n/a	Involved in the study	
☒	☐	ChIP-seq
☒	☐	Flow cytometry
☒	☐	MRI-based neuroimaging

[illegible]
